# Comparative transcriptome analysis of two contrasting watermelon genotypes during fruit development and ripening

**DOI:** 10.1186/s12864-016-3442-3

**Published:** 2017-01-03

**Authors:** Qianglong Zhu, Peng Gao, Shi Liu, Zicheng Zhu, Sikandar Amanullah, Angela R. Davis, Feishi Luan

**Affiliations:** 1 Key Laboratory of Biology and Genetic Improvement of Horticulture Crops (Northeast Region), Ministry of Agriculture, Harbin, Heilongjiang 150030 China; 2 Horticulture College, Northeast Agricultural University, 59 Mucai Street, Harbin, Heilongjiang 150030 China; 3 South Central Agricultural Research Laboratory, Agricultural Research Service, U.S. Department of Agriculture, Currently with HM. Clause 9241 Mace Blvd, Davis, CA 95618 USA

**Keywords:** Watermelon, *Citrullus lanatus*, Fruit ripening, Gene expression, Transcription factors

## Abstract

**Background:**

Watermelon [*Citrullus lanatus* (Thunb.) Matsum. & Nakai] is an economically important crop with an attractive ripe fruit that has colorful flesh. Fruit ripening is a complex, genetically programmed process.

**Results:**

In this study, a comparative transcriptome analysis was performed to identify the regulators and pathways that are involved in the fruit ripening of pale-yellow-flesh cultivated watermelon (COS) and red-flesh cultivated watermelon (LSW177). We first identified 797 novel genes to extend the available reference gene set. Second, 3958 genes in COS and 3503 genes in LSW177 showed at least two-fold variation in expression, and a large number of these differentially expressed genes (DEGs) during fruit ripening were related to carotenoid biosynthesis, plant hormone pathways, and sugar and cell wall metabolism. Third, we noted a correlation between ripening-associated transcripts and metabolites and the key function of these metabolic pathways during fruit ripening.

**Conclusion:**

The results revealed several ripening-associated actions and provide novel insights into the molecular mechanisms underlying the regulation of watermelon fruit ripening.

**Electronic supplementary material:**

The online version of this article (doi:10.1186/s12864-016-3442-3) contains supplementary material, which is available to authorized users.

## Background

Watermelon [*Citrullus lanatus* (Thunb.) Matsum. & Nakai var. lanatus] belongs to the Cucurbitaceae family. According to the latest statistical data from the FAO (http://www.fao.org/faostat/en/), more than 109 million tons of watermelon fruit were produced in 2013, and the production of watermelon fruit accounts for ~9.5% of worldwide vegetable production [1]. The differences in the shape, size, rind thickness and color, flesh texture and color, sugar content, carotenoid content, aroma, flavor, and nutrient composition of the fruit make watermelon an important and well-known component of the daily nutrition of the world’s population and an attractive model of non-climacteric fleshy fruit. The exploration and characterization of the regulatory transcription factors and molecular mechanisms that influence fruit ripening and the formation of attractive characteristics of watermelon fruit would be extremely meaningful for watermelon research and breeding efforts directed at improving this crop.

Fruit ripening is a highly coordinated, genetically programmed and irreversible process involving a series of physiological, biochemical, and organoleptic changes that result in the development of an edible ripe fruit [1, 2]. Fruit development and ripening are regulated by phytohormones, light, temperature, and gene regulation [3]. Numerous studies on fruit ripening in a variety of plant species have suggested that the coordinated expression of a set of genes is a major mechanism influencing fruit ripening. However, the available data regarding the genes associated with fruit growth and ripening in watermelon are limited. Recently, the development and boom of RNA-Seq technology has resulted in its successful application in the analysis of changes in the transcriptome of watermelon fruit. A subtracted and normalized cDNA library representing fruit ripening generated 832 expressed sequence tags (ESTs) [4], and 335 of these were found to be differentially expressed during fruit ripening and were classified into the following ten categories: primary metabolism, amino acid synthesis, protein processing and degradation, membrane and transport, cell division, cytoskeleton, cell wall and metabolism, DNA- and RNA-related gene expression, signal transduction, and defense- and stress-related genes [3]. A digital expression analysis of a larger collection of watermelon ESTs showed that 3023 genes that are differentially expressed during watermelon fruit development and ripening are involved in the Calvin cycle, cellulose biosynthesis, ethylene biosynthesis, glycolysis II and IV, gluconeogenesis, sucrose degradation, the citrulline-nitric oxide cycle, trans-lycopene biosynthesis, β-carotene biosynthesis and flavonoid biosynthesis [5]. After the watermelon genome sequence was published [6], a downstream functional genomics study on the transcriptome of the flesh of cultivated watermelon ’97,103’ and wild watermelon ‘PI296341-FR’ identified 2452 and 322 differentially expressed genes (DEGs) during fruit development, respectively. A gene ontology (GO) analysis of these genes revealed that the biological mechanisms and metabolic pathways associated with fruit value, such as sweetness and flavor, noticeably changed only in the flesh of 97,103 during fruit growth, whereas those associated with abiotic stress were altered primarily in the PI296341-FR flesh [1]. Earlier studies have not yet addressed the question which genes are involved in the process of fruit ripening and the key metabolic pathways important for fruit ripening in cultivated watermelon have not been determined. Furthermore, the gene expression profiles during the development of pale-yellow-flesh watermelon fruit have not been studied. The aim of our study was to comparatively analyze the transcriptomes of two contrasting watermelon genotypes, i.e., red-flesh and pale-yellow-flesh watermelon (LSW177 and COS, respectively), throughout growth during ripening to reveal the genes associated with the development and ripening of *Citrullus lanatus* fruit and to provide further insights for identifying key potential pathways and regulators involved in the development and ripening of cultivated watermelon fruit.

## Results

### Variations in the soluble sugar and lycopene contents during the ripening of COS and LSW177 fruits

**Fig. 1:**
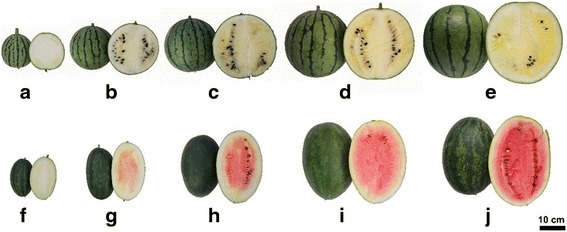
Fruit of watermelon cultivars COS and LSW177 at critical development stages. COS fruit: 10 DAP (**a**), 18 DAP (**b**), 26 DAP (**c**), 34 DAP (**d**), and 42 DAP (**e**). LSW177 fruit: 10 DAP (**f**), 18 DAP (**g**), 26 DAP (**h**), 34 DAP (**i**), and 42 DAP (**j**)

**Fig. 2:**
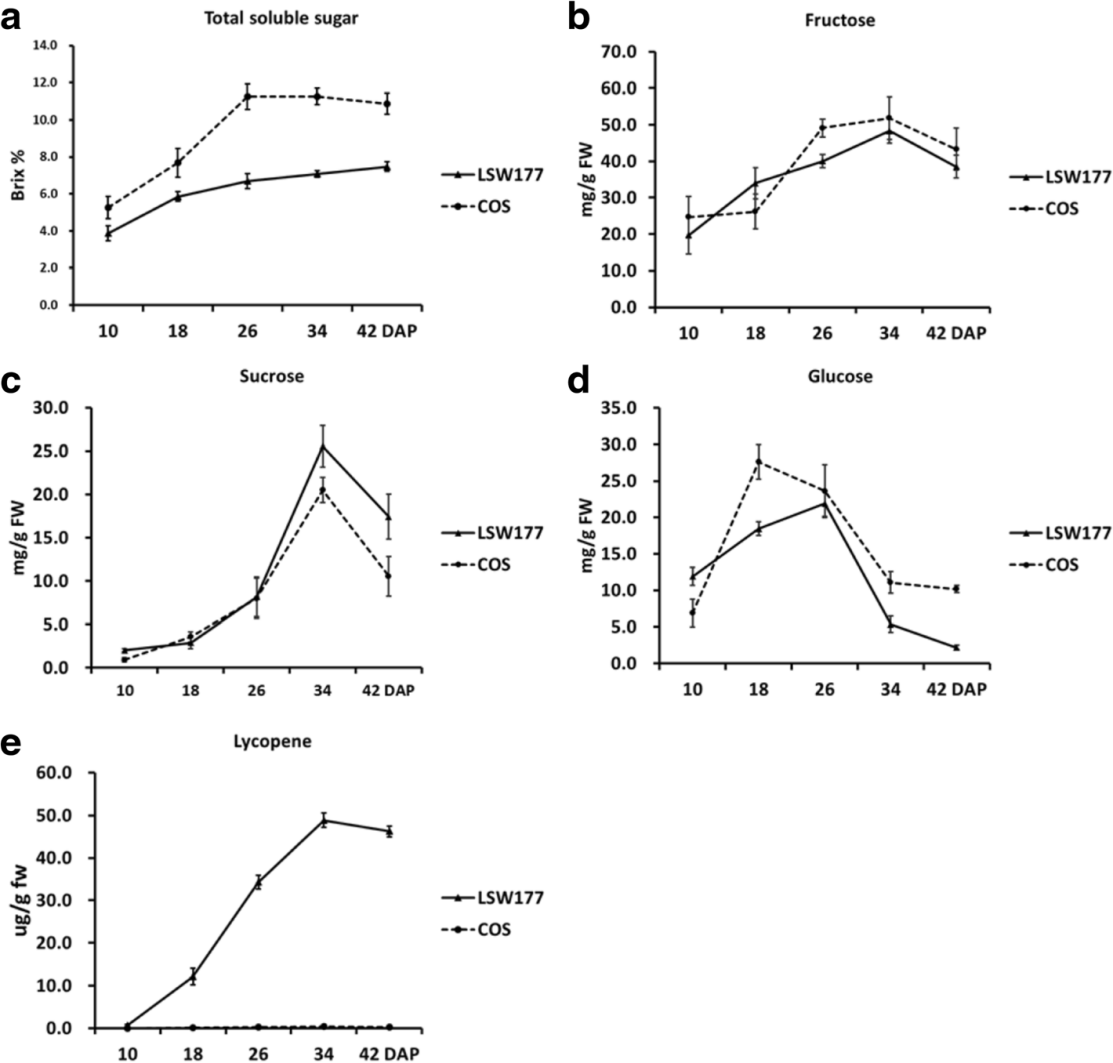
Trends in lycopene and soluble sugar contents in COS and LSW177 fruit during ripening. Lycopene (**a**), total soluble sugar (**b**), glucose (**c**), fructose (**d**), and sucrose (**e**) were extracted at 10, 18, 26, 34, and 42 DAP. Three individual replicates were used to reduce the experimental error. The bars represent the standard error (SE) (*n* = 3)

The soluble sugar and lycopene contents of watermelon fruit largely determine its quality. Hence, the soluble sugar and lycopene contents of COS and LSW177 were measured during fruit ripening. Previous reports have emphasized the existence of different maturation stages regarding flesh quality. Immature white flesh, white-pink flesh, red flesh, and full-ripe (10, 18, 26, and 34 days after pollination [DAP], respectively) are the four critical ripening stages of red-flesh cultivated watermelon [1, 5, 7]. To obtain insights into the development of watermelon fruit, we included an over-ripening stage (42 DAP) in addition to the other four stages in our experiments analyzing the ripening of watermelon fruit (Fig. 1). In ripened watermelon fruit, the dominant soluble sugars are sucrose, fructose, and glucose. The trends of the changes in the soluble sugar contents are shown in Fig. 2. The total soluble sugar (TSS), sucrose, and fructose contents peaked during fruit ripening but decreased during over-ripening in both COS and LSW177 (Fig. 2a-c). The TSS content in COS was markedly higher than that in LSW177 during fruit ripening (Fig. 2a). From 26 to 42 DAP, the fructose concentration in COS was higher than that in LSW177 (Fig. 2b), whereas the sucrose content in COS was lower than that in LSW177 (Fig. 2c). In addition, the glucose content peaked at the early stage of fruit ripening in the two cultivars and was rapidly restored to the baseline value during the period from 18 DAP in COS and 26 DAP in LSW177 to 42 DAP (Fig. 2d). Moreover, the glucose content in COS was higher than that in LSW177 from 18 to 42 DAP. Notably, the lycopene content in LSW177 significantly increased during fruit ripening and decreased slightly during over-ripening (Fig. 2e), whereas the lycopene content in COS was markedly lower than that in LSW177 and changed steadily from 18 to 42 DAP. These findings suggest that the qualities of COS and LSW177 fruits are significantly different during fruit development and ripening.

### Sequencing and transcript assembly identify novel genes expressed in watermelon during fruit ripening

In a recent study [8], we characterized the carotenoid contents in COS and LSW177, and these two cultivars were selected for further study due to their different lycopene contents and the degree of difference in their mechanisms regulating lycopene accumulation during fruit ripening. A total of 20 cDNA libraries prepared from fruit flesh samples at the four critical ripening stages and one over-ripening stage (with two biological replicates for each stage and watermelon species) were sequenced (described in methods; Fig. 1). The raw sequencing data were assessed for quality and subjected to data filtering, and 859 million clean paired-end reads of 125 bp in length were obtained for further analysis. All of the clean reads were deposited in the NCBI Short Read Archive (SRA) database under the accession numbers SRX2037189 and SRX2037303.

The fragments were mapped to the high-quality watermelon reference genome [6] using TopHat [9, 10]. A total of 763 million reads were aligned to the reference genome, yielding an overall mapping percentage of 88.7% with a standard deviation of 5.3% (Table 1). Ultimately, 24,237 genes with 63,167 transcripts were identified by Cufflinks and used as reference transcripts to determine the read count using HTSeq-count.

**Table 1:** Number of clean reads generated from each sample were sequenced and mapped to the 97103 genome using TopHat

Sample name	Total no. of clean reads	Reads mapped	Percentage of mapped reads (%)
C10_R1	45,512,554	41,874,167	92.0%
C10_R2	44,710,296	40,648,859	90.9%
C18_R1	40,934,174	34536,742	84.4%
C18_R2	37,227,820	33,324,089	89.5%
C26_R1	34,522,116	31,548,790	91.4%
C26_R2	56,624,960	50,605,220	89.4%
C34_R1	41,100,524	37,558,563	91.4%
C34_R2	41,191,050	37,844,289	91.9%
C42_R1	42,167,238	38,172,566	90.5%
C42_R2	41,281,894	37,498,029	90.8%
L10_R1	41,457,238	37,607,060	90.7%
L10_R2	39,140,418	35,380,533	90.4%
L18_R1	39,760,896	35,735,866	89.9%
L18_R2	36,210,552	32,394282	89.5%
L26_R1	64,791,768	59,029,106	91.1%
L26_R2	41,861,552	37,817,640	90.3%
L34_R1	37,945,012	26,104,240	68.8%
L34_R2	44,288,670	39,169,275	88.4%
L42_R1	46,102,036	38,075,275	82.6%
L42_R2	41,935,016	38,165,978	91.0%

The expression data generated in our study improve the previous annotations of the watermelon genome, which has 23,440 predicted genes [6]. Genome-guided assemblies were performed to serve as sequence verification for transcriptome re-annotation in watermelon fruit during ripening. We identified 797 novel genes corresponding to 2057 transcripts with a typical length of 2535 bp (see Additional file 1), and these identifications mainly resulted from the unknown intergenic transcripts and the opposite strands of the annotated genes. These novel genes were functionally annotated by aligning the gene sequences to the NCBI non-redundant (Nr) [11], SwissProt [12], GO [13], and Kyoto Encyclopedia of Genes and Genomes (KEGG) [14] protein databases (e-value < 1e-5) by BLASTX to identify the proteins with the same peak sequence as the compatible novel genes (see Additional file 2). Ultimately, 91.3, 60.2, 40.3 and 14.4% of the novel genes were successfully annotated in the four protein databases, respectively.

### DEGs analysis of COS and LSW177 during fruit ripening

**Fig. 3:**
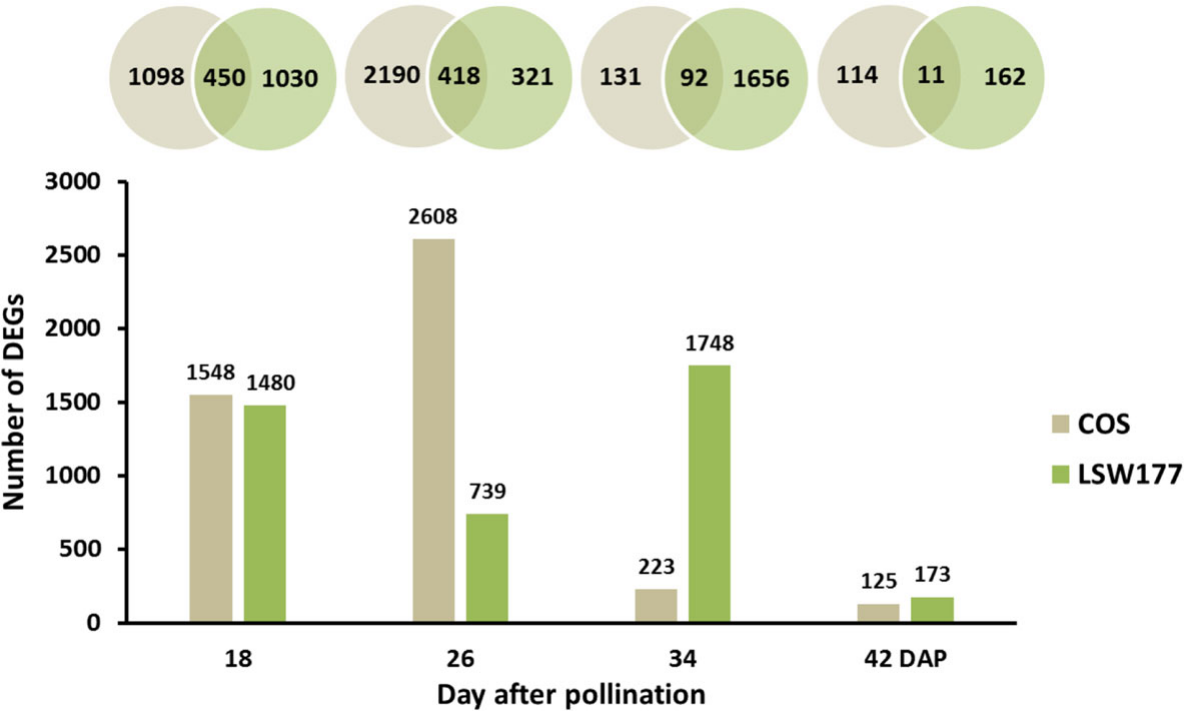
Distribution of differentially expressed genes (DEGs) on different days after pollination during watermelon fruit development and ripening. Overlap in the Venn diagram indicates that the DEGs appeared in both samples represented by the circles. The bar chart represents the distribution of DEGs in different samples. Light green and light brown represent the DEGs in COS and LSW177, respectively

To categorize the DEGs during fruit ripening, we used a stringent value of FDR ≤ 0.05 and an absolute value of log_2_ Ratio ≥ 1 as the thresholds for identifying significant differences in gene expression between two close stages (the earlier stage was considered the control sample, and the later stage was the treated sample) during fruit ripening. As a result, 3958 developmental DEGs in COS and 3503 developmental DEGs in LSW177 were obtained for further analysis (Additional files 3 and 4). The DEGs in COS and LSW177 were further analyzed at each stage during fruit ripening (Fig. 3). At 18 DAP, 1548 and 1480 genes were differentially expressed in COS and LSW177, respectively, whereas only 450 genes were differentially expressed in both cultivars. However, at 26 DAP, 2608 DEGs were detected in COS, and this peak in the number of DEGs in COS revealed the significance of this period. The number of DEGs at later time points was markedly lower than that in COS at 26 DAP. Over time, the number of DEGs in COS markedly decreased to 223 at 34 DAP and 125 at 42 DAP, indicating that the fruit growth rate of COS started to slow down and that the fruit was already ripe or in the over-ripening state. In contrast, the number of DEGs in LSW177 did not peak until 34 DAP and then decreased significantly to 173 at 42 DAP, which suggests that the duration of the mature stage of LSW177 was longer than that of COS. In addition, the analysis identified few DEGs that were differentially expressed in both cultivars.

### Verification of the expression of some DEGs detected during fruit ripening

Quantitative real-time PCR (qPCR) analysis was performed to validate our transcriptome profiling dataset of procured genes by correlating their qPCR results with standard data from the RNA-Seq analysis (presented in the Methods). We observed clear positive correlations between the qPCR and RNA-Seq data for these two cultivars at the overall fruit ripening stages (Additional file 5: Figure S1). Statistical analysis indicated that the disparity between the qPCR and RNA-Seq results depended on the expression levels of the genes under study. Hence, for genes with very low or high expression levels, qPCR verification was less reliable.

### GO term analysis of DEGs

**Fig. 4:**
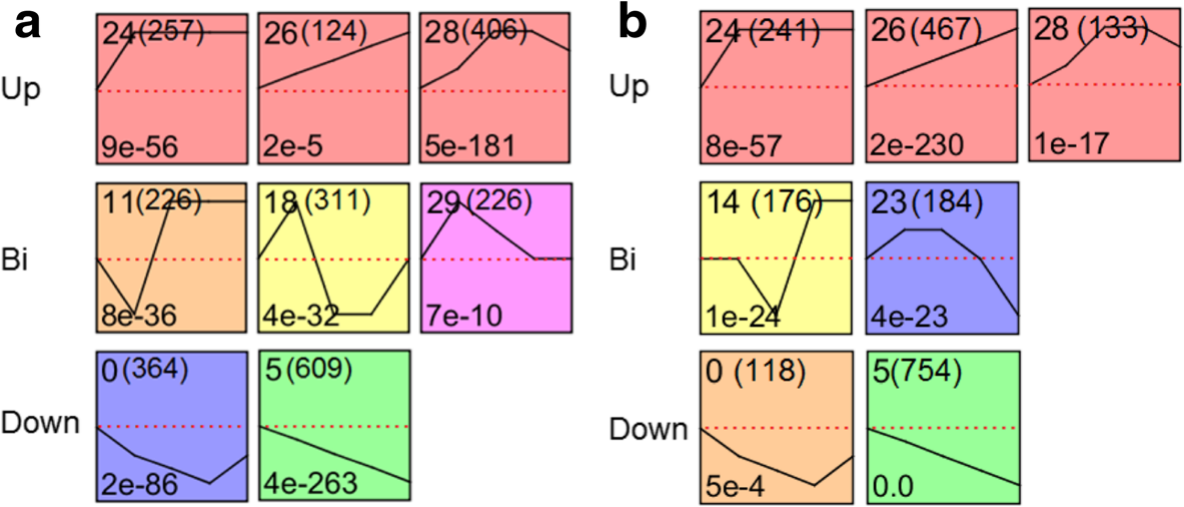
Significantly enriched profiles (*P* value ≤ 0.05) during fruit ripening as revealed by time-course analysis. Profiles in COS (**a**) and LSW177 (**b**). The profiles were classified into three groups, Up (upregulated), Bi (biphasic expression pattern), and Down (downregulated), and further ordered based on their profile number following the number of genes in the bracket (top left-hand corner). The *P* value assigned to each profile is shown in the bottom left-hand corner. Significantly different profiles are represented by different background colors

To examine the expression profiles of the identified DEGs, 3375 DEGs from COS and 2835 DEGs from LSW177 were clustered into 32 profiles by Short Time-Series Expression Miner (STEM) [15]. Specifically, 2523 DEGs from COS were clustered into eight profiles (*P* value ≤ 0.05), including two types of downregulated patterns (Profile 0 and Profile 5), three upregulated patterns (Profile 24, Profile 26 and Profile 28), and three biphasic expression patterns (Profile 11, Profile 18, and Profile 29) (Fig. 4a), whereas 2073 DEGs from LSW177 were clustered into seven profiles (*P* value ≤ 0.05), including two downregulated patterns (Profile 0 and Profile 5), three upregulated patterns (Profile 24, Profile 26 and Profile 28), and two biphasic expression patterns (Profile 14 and Profile 23) (Fig. 4b). The DEGs within the up- and downregulated cluster groups established for COS and LSW177 were then subjected to GO term analysis (Additional file 5: Figure S2A-2B) and allocated into three core categories, e.g., cellular component, biological process, and molecular function. Within the cellular component category, a significant number of upregulated and downregulated DEGs were divided into cell, cell parts and organelles. Within the biological process category, most of the DEGs were classified into cellular process and metabolic process. Within the molecular function category, catalytic activity and binding were the subcategories containing the most DEGs.

### KEGG pathway enrichment analysis of DEGs

**Fig. 5:**
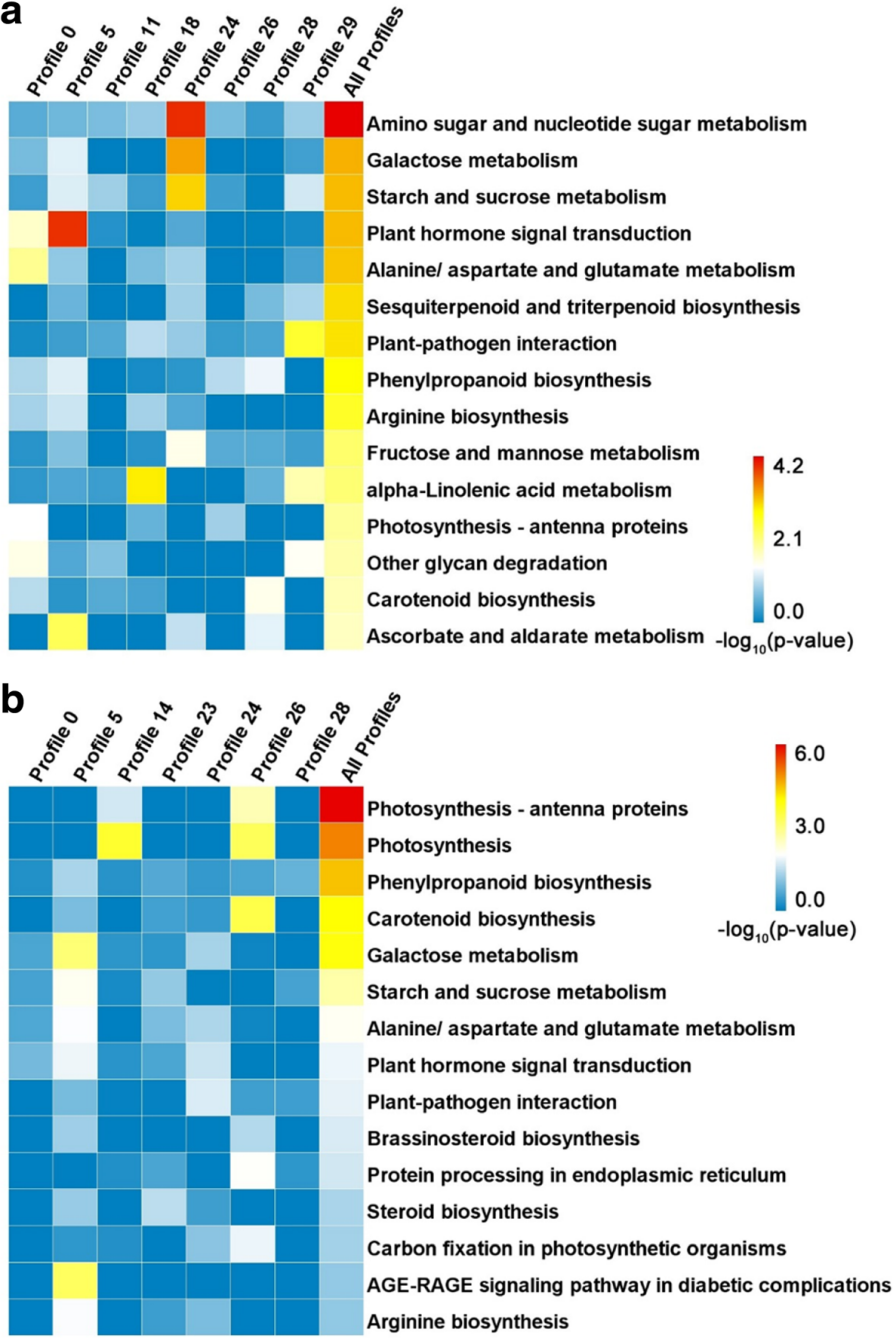
Top 15 KEGG pathways sorted by *P* value for annotating DEGs in COS and LSW177. Of the DEGs identified in COS (**a**) and LSW177 (**b**) from KEGG pathway enrichment analysis for genes in different clusters, the top 15 pathways were selected according to the KO annotation for all DEGs involved in fruit ripening in COS or LSW177. Fisher’s exact test was used to identify the significance of the pathway in each profile (P) compared to the whole-transcriptome background. The *P* values were converted to -log10 and are presented in a heat map. Deeper color represents a higher degree of pathway enrichment

The DEGs in COS and LSW177 were subjected to a KEGG pathway enrichment analysis, and 17.7% (700/3958) of the DEGs in COS could be annotated into 119 different metabolic pathways (Additional file 6). Figure 5a shows the top 15 most significantly enriched metabolic/biological pathways with annotation for each highly represented profile in COS. In contrast, 18.2% (638/3503) of the DEGs in LSW177 could be assigned to 115 different metabolic pathways (Additional file 6), and the 15 top KEGG pathways with the most representation are shown in Fig. 5b. Of these KEGG pathways, galactose metabolism (ko00052), starch and sucrose metabolism (ko00500), plant hormone signal transduction (ko04075), alanine, aspartate and glutamate metabolism (ko00250), plant-pathogen interaction (ko04626), phenylpropanoid biosynthesis (ko00940), arginine biosynthesis (ko00220); photosynthesis-antenna proteins (ko00196), and carotenoid biosynthesis (ko00906) were the KEGG pathways identified in both COS and LSW177. Notably, more DEGs in LSW177 during fruit ripening than in COS were significantly enriched in carotenoid biosynthesis (*P* value = 2.4E-5 in LSW177; *P* value = 1.2E-2 in COS), whereas the DEGs in COS were more significantly involved in pathways associated with plant hormone signal transduction (*P* value = 7.2E-3 in LSW177; *P* value = 4.4E-4 in COS) and starch and sucrose metabolism (*P* value = 8.2E-4 in LSW177; *P* value = 4.4E-4 in COS). Profile 5 contained mostly DEGs from the eight highly represented profiles in COS, and their expression was consistently downregulated during fruit ripening. Genes involved in plant hormone signal transduction and ascorbate and aldarate metabolism were significantly enriched in Profile 5. The enriched categories of the DEGs in Profile 24 mainly included amino sugar and nucleotide sugar metabolism, galactose metabolism, and starch and sucrose metabolism; the expression of these genes increased at the initial stage and was unchanged during fruit ripening. The expression profile of 28 clusters, including the enriched categories of phenylpropanoid biosynthesis, carotenoid biosynthesis, and ascorbate and aldarate metabolism, increased by 26 DAP and gradually decreased from 32 to 42 DAP during fruit ripening. The expression of the genes in Profile 26 of LSW177 consistently increased during fruit ripening, and this profile was enriched in genes involved in carotenoid biosynthesis and photosynthesis. With the highest degree of functional enrichment, Profile 5 contained approximately half of the enriched categories in the seven highly represented profiles in LSW177, including genes involved in galactose metabolism, starch and sucrose metabolism, plant hormone signal transduction, and carotenoid biosynthesis.

### An integrative analysis of DEGs during fruit ripening revealed the key pathways involved in the ripening of cultivated watermelon fruit

**Fig. 6:**
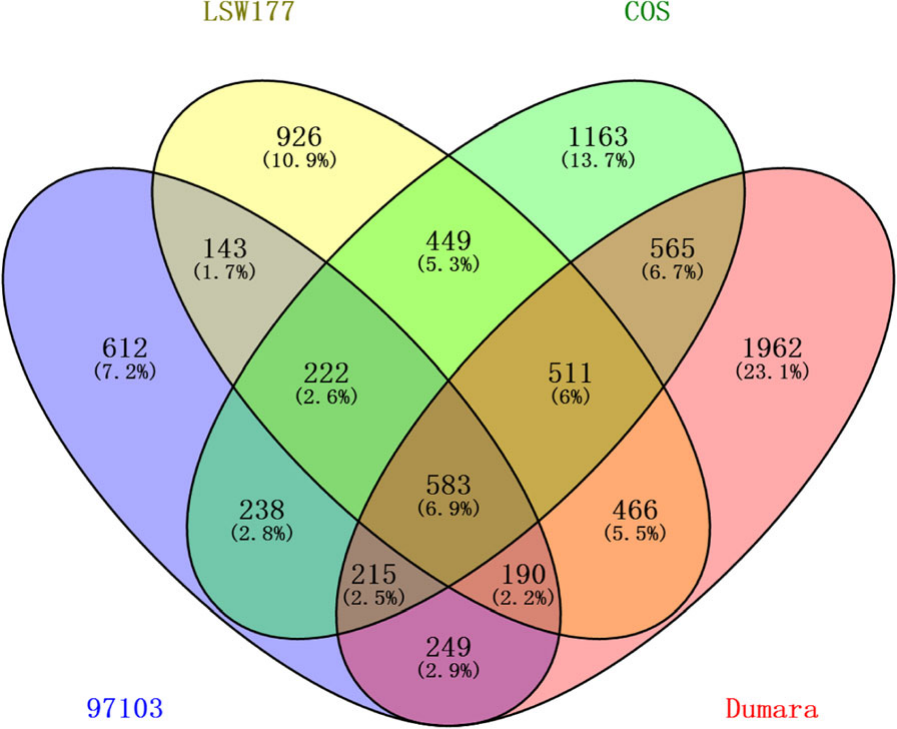
Comparison of the DEGs detected in four watermelon cultivars (COS, LSW177, 97103, and Dumara) during fruit ripening

**Fig. 7:**
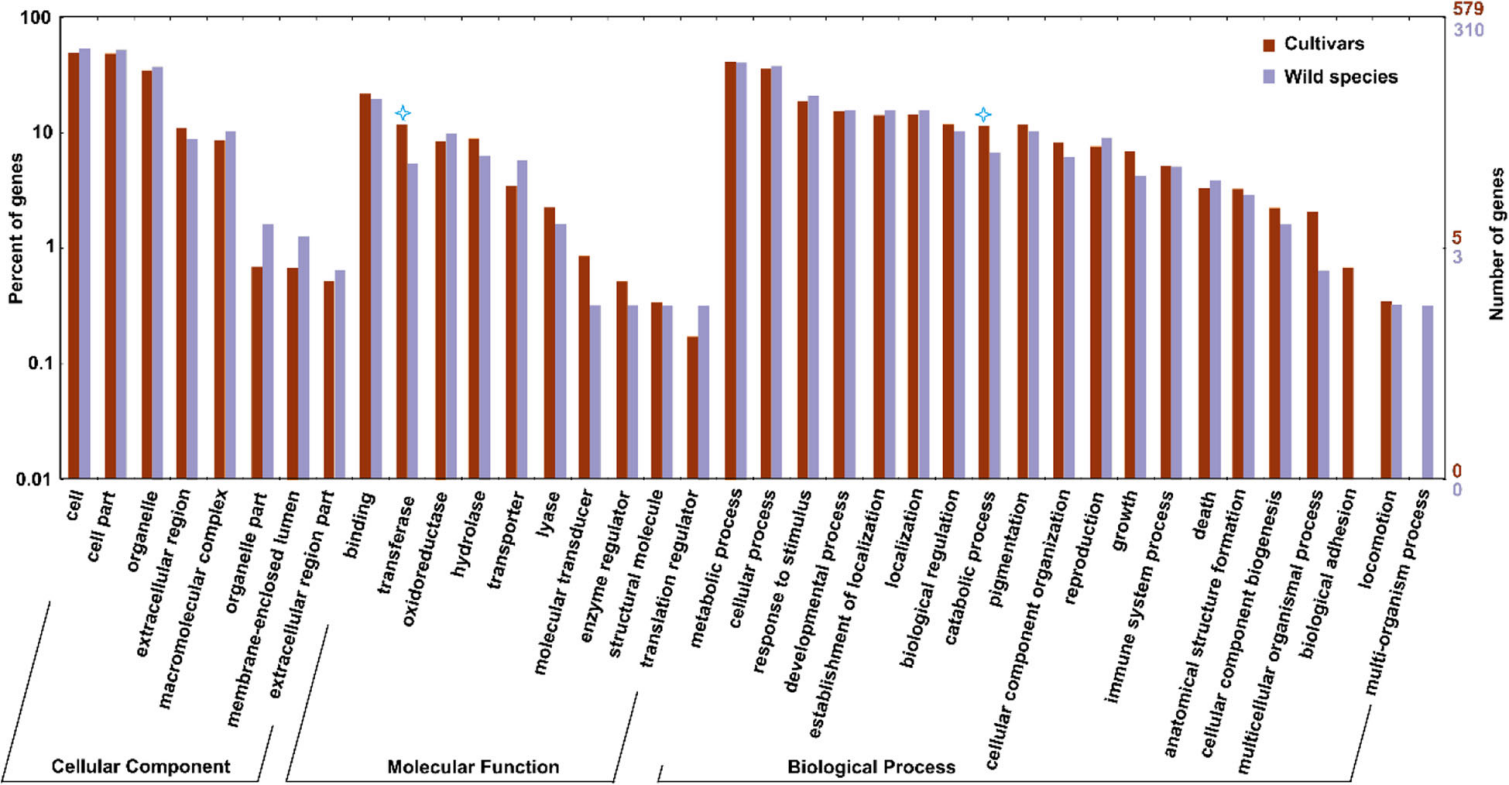
GO classification of the DEGs detected in four watermelon cultivars (COS, LSW177, 97103, and Dumara) and wild species (PI296341-FR) during fruit ripening. The blue star indicates statistically significant differences (*P* value < 0.05) analyzed using the Chi-square test

To reveal the key pathways involved in the ripening of cultivated watermelon fruit, we compared the DEGs from four cultivars: COS (3958 DEGs) and LSW177 (3503 DEGs) in this study and Dumara (4756 DEGs) and 97103 (2452 DEGs) in previous studies [1, 7]. We found that 583 DEGs overlapped (Fig. 6) and used these as fruit-ripening-responsive genes to identify the key pathways during fruit ripening while avoiding the genotype × environment effect, which exhibits variations in different watermelon cultivars. A total of 322 DEGs during fruit ripening in the wild species PI296341-FR were used to represent key genes involved in fruit ripening. GO categories were assigned to these groups of 583 and 322 DEGs. Figure 7 shows the assigning of GO terms according to the equivalent biological process, molecular role and cellular component. We noted that more DEGs were significantly enriched in the categories of transferase activity (*P* value < 0.05, Chi-square test) and catabolic activity (*P* value < 0.05) in the cultivars than in the wild species. A KEGG analysis assigned the DEGs from the cultivars and wild species to 76 and 44 metabolic pathways, respectively. The entire list of metabolic pathways is provided in Additional file 7. The top 20 significantly enriched KO pathways in the cultivars sorted by *P* value (Fisher’s exact test) and their corresponding enrichment in the wild species are presented in Table 2. Interestingly, 583 DEGs were significantly enriched in 10 KO pathways (*P* value < 0.05), namely phenylpropanoid biosynthesis (ko00940), galactose metabolism (ko00052), other glycan degradation (ko00511), carotenoid biosynthesis (ko00906), arginine biosynthesis (ko00220), monobactam biosynthesis (ko00261), brassinosteroid biosynthesis (ko00905), pentose and glucuronate interconversions (ko00040), plant hormone signal transduction (ko04075), and alanine, aspartate and glutamate metabolism (ko00250) (Table 2). However, compared with the cultivars, the 322 DEGs in the wild species were less significantly enriched in these pathways, with the exception of arginine biosynthesis and alanine, aspartate and glutamate metabolism (Table 2). Interestingly, four of the ten metabolic pathways were annotated to relate to sugar metabolism and cell wall metabolism, including galactose metabolism, pentose and glucuronate interconversions, other glycan degradation, and phenylpropanoid biosynthesis, but none of these was significant during fruit ripening in the wild watermelon species. These differences might suggest that with the domestication and improvement of wild watermelon species, watermelon fruit flesh with a high utilization ratio of carbohydrates, stronger sugar-mediated signaling, and greater sucrose accumulation would be selected by humans [6], which would increase the soluble sugar content and improve the appearance of the fruit flesh. In addition, we noted that carotenoid accumulation (*P* value < 0.05) in the wild species was also significantly enriched in DEGs, suggesting that carotenoid biosynthesis is more important than other metabolic pathways in watermelon fruit flesh ripening.

**Table 2:** Top 20 KEGG pathways of significantly enriched DEGs in four watermelon cultivars (COS, LSW177, 97103, and Dumara) sorted by –Log_10_ *P* value (Fisher's exact test) and compared with a wild species (PI296341-FR)

Pathway	Cultivars		Wild		Pathway ID
	Gene number	–Log_10_ *P*	Gene number	–Log_10_ *P*	
Phenylpropanoid biosynthesis	15	4.8 ^a^	5	0.9	ko00940
Galactose metabolism	6	2.6 ^a^	1	0.2	ko00052
Other glycan degradation	3	2.1 ^a^	-	-	ko00511
Carotenoid biosynthesis	4	2.0 ^a^	3	1.8 ^a^	ko00906
Arginine biosynthesis	4	1.8 ^a^	4	2.5 ^a^	ko00220
Monobactam biosynthesis	2	1.7 ^a^	-	-	ko00261
Brassinosteroid biosynthesis	2	1.6 ^a^	-	-	ko00905
Pentose and glucuronate interconversions	7	1.5 ^a^	2	0.2	ko00040
Plant hormone signal transduction	12	1.4 ^a^	8	1.2	ko04075
Alanine, aspartate and glutamate metabolism	4	1.3 ^a^	5	2.8 ^a^	ko00250
Lysine biosynthesis	2	1.2	-	-	ko00300
Cysteine and methionine metabolism	5	1.1	2	0.4	ko00270
Tryptophan metabolism	3	1.1	-	-	ko00380
Biosynthesis of amino acids	9	1.0	4	0.3	ko01230
Plant-pathogen interaction	10	1.0	3	0.1	ko04626
Carbon fixation in photosynthetic organisms	4	1.0	1	0.2	ko00710
Glycine, serine and threonine metabolism	4	1.0	-	-	ko00260
Alpha-linolenic acid metabolism	3	0.9	2	0.7	ko00592
Carbon metabolism	9	0.9	3	0.1	ko01200
Linoleic acid metabolism	2	0.8	-	-	ko00591

^a^Significantly enriched pathway with *P* value < 0.05

### DEGs in carotenoid biosynthesis, plant hormone signal transduction, and sugar and cell wall metabolism during the ripening of COS and LSW177 fruit

**Fig. 8:**
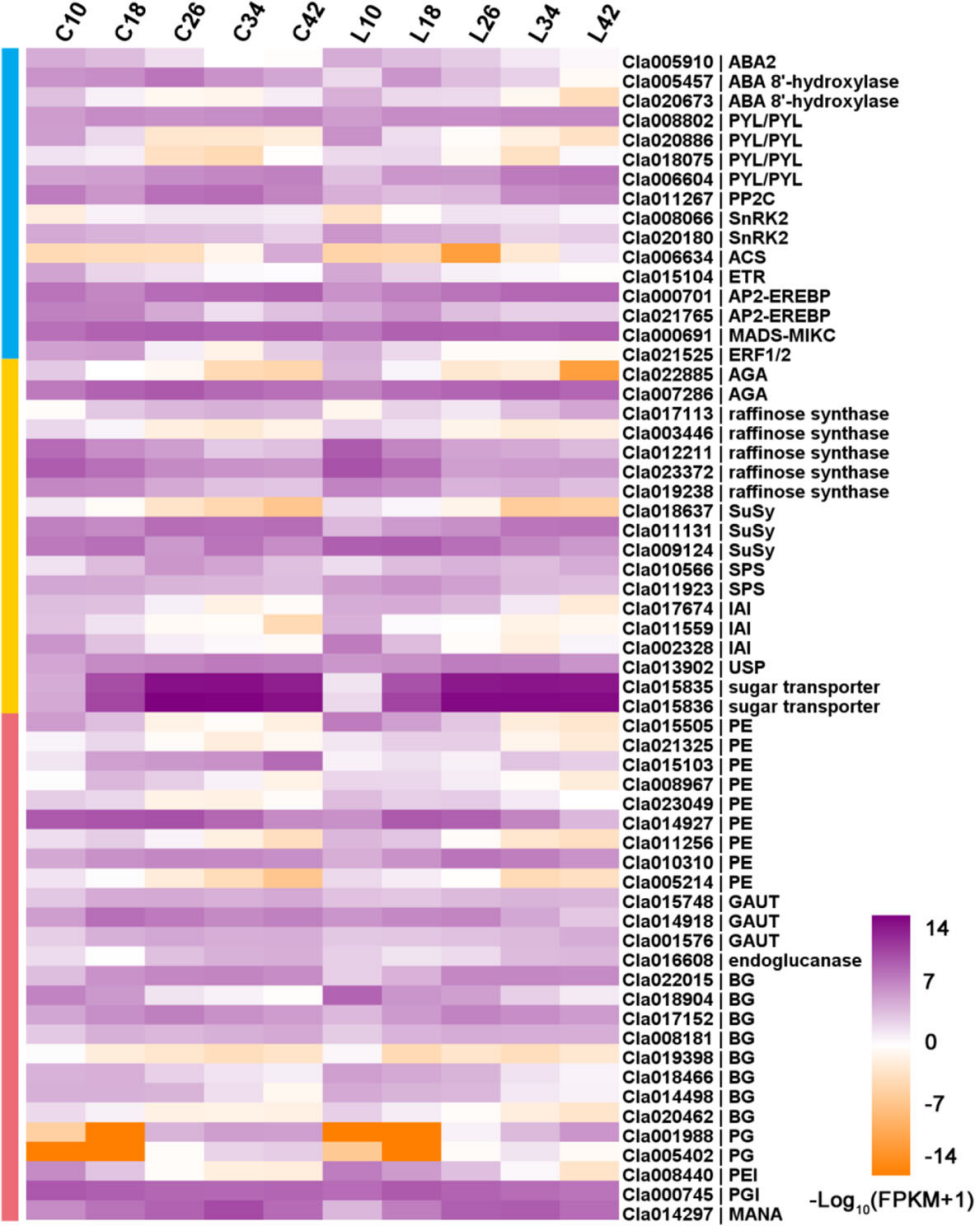
Heat map diagram of the expression levels of DEGs. Gene expression data were normalized to log10. The DEGs are involved in plant hormone biosynthesis and signaling transduction (*blue*), sugar metabolism (*red*), and cell wall metabolism (*green*)

The numbers of DEGs involved in carotenoid biosynthesis, plant hormone signal transduction, sugar metabolism and cell wall metabolism during fruit ripening are listed in Table 3. A total of nine DEGs in LSW177 were associated with the carotenoid biosynthesis pathway, and seven of these, which encoded phytoene synthase (*PSY*: *Cla009122*), ζ-carotene isomerase (*Z-ISO*: *Cla010839*), ζ-carotene desaturase (*ZDS*: *Cla003751*), β-carotene 3-hydroxylase (*CHYB*: *Cla006149* and *Cla011420*), and 9-cis-epoxycarotenoid dioxygenase (*NCED*: *Cla009779*), were clustered into Profile 24, Profile 26 or Profile 28, showing upregulated trends. Only *Cla003169* encoding PSY was downregulated (Profile 0 and Profile 5). In contrast, of the six DEGs in COS, only three, encoding PSY (*Cla009122*), CHYB (*Cla006149*), and NCED (*Cla009779*), were upregulated (Profile 24, Profile 26, and Profile 28), two showed biphasic expression patterns (Profile 11, Profile 18, and Profile 29), and one was downregulated (Profile 0 and Profile 5). These DEGs encoded ζ-ring hydroxylase (*LUT5*: *Cla000655*) and NCED (*Cla005404* and *Cla005453*). Some of the DEGs involved in plant hormone formation and signal transduction, particularly the biosynthesis and signal transduction of ABA and ethylene, displayed a different expression pattern (Fig. 8); for example, the expression of 1-aminocyclopropane-1-carboxylate synthase (*ACS*: *Cla006634*) and serine/threonine-protein kinase (*SnRK2*: *Cla008066*) in COS peaked during fruit ripening, whereas the expression of *ABA 8-hydroxydase* (*Cla020673*), xanthoxin dehydrogenase (*ABA2*: *Cla005910*), abscisic acid receptor PYR/PYL family (*PYR/PYL*: *Cla020886*), and ethylene receptor (ETR: *Cla015104*) in COS decreased during fruit ripening, and that of ethylene-responsive TF (*ERF*: *Cla021525*) in COS showed a biphasic expression pattern. The expression of an *ABA 8’-hydroxylase* (*Cla005457*) and two *PYR/PYLs* (*Cla008802* and *Cla006604*) in LSW177 increased during fruit ripening, whereas the expression of *ABA2* (*Cla005910*), an *ABA 8’-hydroxylase*, a *PYR/PYL* (*Cla020886*), an *SnRK* (*Cla020180*), and an *ETR* (*Cla015104*) in LSW177 decreased during fruit ripening. Several of the DEGs involved in sugar metabolism and cell wall metabolism, involving genes encoding two α-galactosidases (*AGA*: *Cla022885* and *Cla007286*), five raffinose synthases (*Cla017113*, *Cla003446*, *Cla012211*, *Cla023372*, and *Cla019238*), three sucrose synthases (*SuSy*: *Cla018637*, *Cla011131*, and *Cla009124*), two sucrose-phosphate synthases (*SPS*: *Cla010566* and *Cla011923*), two insoluble acid invertases (*IAI*: *Cla017674* and *Cla002328*), UDP-sugar pyrophosphorylase (*USP*: *Cla013902*), two sugar transporters (*Cla015835* and *Cla015836*), three α-1,4-galacturonosyltransferases (*GAUT*: *Cla015748*, *Cla014918*, and *Cla001576*), nine pectinesterases (*PE*: *Cla015505*, *Cla021325*, *Cla015103*, *Cla008967*, *Cla023049*, *Cla014927*, *Cla011256*, *Cla010310*, and *Cla005214*), α-mannosidase (*MANA*: *Cla014297*), endoglucanase (*Cla016608*), and eight β-glucosidases (*BG*: *Cla022015*, *Cla018904*, *Cla017152*, *Cla008181*, *Cla019398*, *Cla018466*, *Cla014498*, and *Cla020462*), also underwent major modifications during fruit ripening (Fig. 8). Notably, the two insoluble acid invertases were downregulated in both cultivars, α-mannosidase and two sugar transporters were upregulated in both cultivars, and the expression patterns of the other DEGs in COS and LSW177 presented differences.

**Table 3:** List of some of the important differentially expressed genes between the different ripening stages in COS and LSW177

Components	COS				LSW177			
	All	Up	Bi	Down	All	Up	Bi	Down
Carotenoid biosynthesis								
PSY	1	1	0	0	2	1	0	1
Z-ISO	0	0	0	0	1	1	0	0
ZDS	0	0	0	0	1	1	0	0
CHYB	1	1	0	0	2	2	0	0
LUT5	1	0	1	0	0	0	0	0
ZEP	0	0	0	0	1	1	0	0
NCED	3	1	1	1	2	1	0	0
Plant hormone biosynthesis and signal transduction								
ABA2	1	0	0	1	1	0	0	1
ABA8'-hydroxylase	1	0	0	1	2	1	1	0
ACS	1	1	0	0	0	0	0	0
PYL	2	0	0	2	3	2	0	1
PP2C	2	0	0	0	3	0	1	0
SnRK2	2	1	0	0	1	0	0	1
ETR	1	0	0	1	1	0	0	1
ERF	1	0	1	0	0	0	0	0
Sugar metabolism and cell wall metabolism								
AGA	2	0	0	1	1	1	0	0
Raffinose synthesis	3	1	0	2	6	1	0	4
SuSy	2	0	1	0	3	1	0	2
SPS	1	1	0	0	1	0	0	1
IAI	2	0	0	2	2	0	0	2
Sugar transporter	2	2	0	0	2	2	0	0
GAUT	3	3	0	0	0	0	0	0
PE	5	1	2	2	6	1	3	2
BG	8	2	0	4	8	2	0	3
Endoglucanase	1	0	1	0	1	0	0	0
MANA	1	1	0	0	1	1	0	0

### Analysis of TFs involved in watermelon fruit development and ripening

**Fig. 9:**
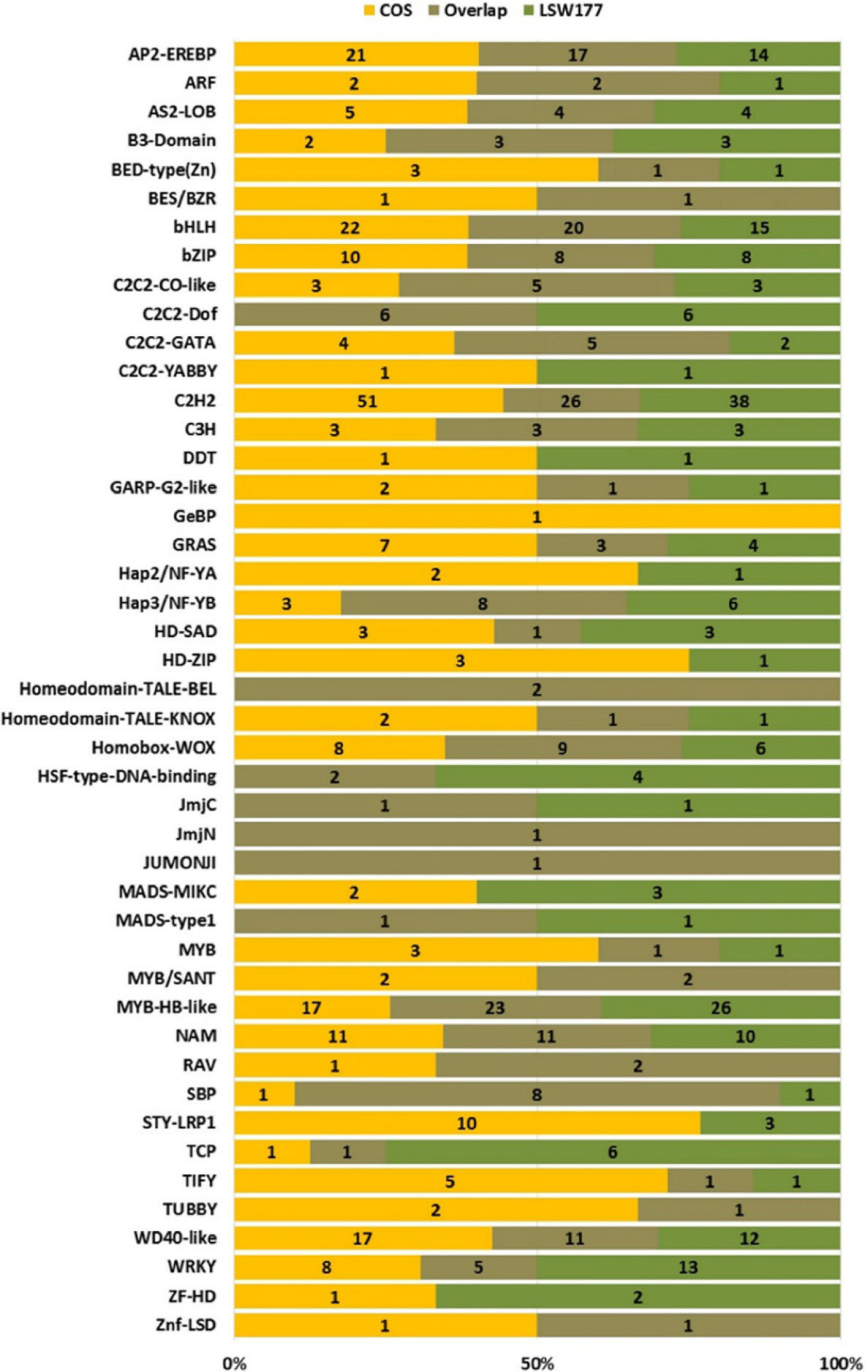
Characterization of the transcription factors of the DEGs during fruit ripening. Distribution of differentially expressed TF families between COS and LSW177

By modulating gene transcription at precise times and during distinct processes, TFs are activated upon wounding, physiological illnesses and internal or external stimulation [16, 17]. To determine which TF families play vital roles in the development and maturation of watermelon fruit, the DEGs in COS and LSW177 were annotated and classified as TFs using PlantTFcat [18]. From these DEGs, 427 TFs in COS and 404 TFs in LSW177 were identified (Additional file 8). In general, 648 non-overlapping putative TFs were further classified into 45 TF families that were present in the PlantTFcat database (Fig. 9). Of these differentially expressed TFs, *AP2-ERFBP*, *bHLH*, *C2H2*, and *MYB-HB-like* were the most abundant in the two cultivars and have been identified and implicated in many diverse functions described in this database, including hormone signal transduction, cell proliferation, protein-protein interactions, anthocyanin biosynthesis, and fruit dehiscence, which are involved in the development and ripening of fruit in normal or reverse form.

## Discussion

**Fig. 10:**
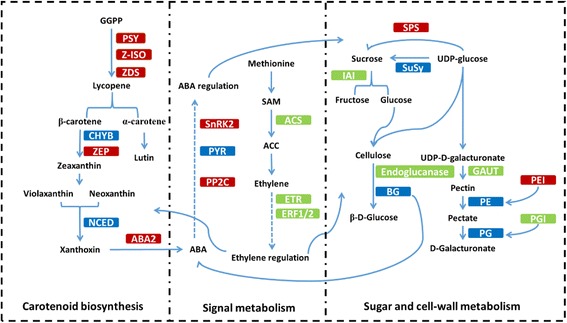
Some of the biological pathways involved in watermelon fruit ripening. Red boxes indicate genes that are upregulated in LSW177 compared to COS, green boxes indicate genes that are downregulated in LSW177 compared to COS, and blue boxes indicate genes that exhibit mixed expression patterns (both up- and downregulated) in LSW177 compared to COS

Fruit ripening is a broadly used, genetic and irreversible process that contributes to a chain of physiological, biochemical and sensory changes that result in the development of soft, mature, high-quality fruits [1, 19]. RNA-Seq technology was used to reveal the key roles of metabolic pathways during the ripening of cultivated watermelon fruit and to explore the transcriptomic differences between two contrasting cultivated watermelon genotypes. A total of 3958 DEGs in COS and 3503 DEGs in LSW177 were identified to reveal a group of genes that contribute to the development and maturation of these two watermelon cultivars. In addition, 583 DEGs in four watermelon cultivars during fruit ripening were identified through an integrative transcriptome analysis. Based on a gene functional enrichment analysis, these DEGs were combined with public data and isolated to identify the most important pathways involved in fruit ripening. In addition to the extensively enriched pathways in COS and LSW177, some DEGs were found to be involved in carotenoid formation, plant hormone signal transduction, sugar metabolism and cell wall metabolism and might have unique functions in cultivated watermelon during fruit ripening. These metabolic pathways are also important for fruit ripening in melon [20], tomato [21] and orange [19]. These pathways have the ability to create an organized metabolic association that possibly cooperates during fruit ripening in cultivated watermelon. Several of the regulated genes in these pathways are included in Fig. 10. The obtained evidence provides a detailed picture of the regulatory complex that contributes to the ripening of cultivated watermelon fruit and reveals transcriptomic differences between COS and LSW177 fruits.

Carotenoids are a diverse group of colorful tints that occur naturally and are fundamental in plants, where they play a pivotal role regarding human nutrition and health benefits [22]. Carotenoid formation is monitored throughout the lifespan of a plant and changes according to developmental necessity and in response to external environmental stimuli. The carotenoid formation pathway initiates with the synthesis of phytoene via geranylgeranyl diphosphate (GGPP) in the innermost isoprenoid pathway. Phytoene is further metabolized through desaturations, cyclizations and hydroxylations to yield various products, such as lycopene, carotenes and xanthophylls, through a sequence of tandem reactions. The most important carotenoid accumulated in red-flesh watermelon is lycopene, and its typical level is approximately 60%, which is more than that found in tomato fruit [23]. The predominant carotenoids in canary-yellow and pale-yellow phenotypes is zeaxanthin, neoxanthin, violaxanthin and neochrome [24]. There is a variety of strategies for organizing carotenoid biosynthesis and accumulation in plant tissues [7]; environmental signaling, plastid compartment size, and post-transcriptional regulation control carotenoid formation and accumulation, but the transcriptional regulation of carotenoid gene expression is considered a key mechanism through which the biosynthesis of peculiar carotenoids is organized during fruit ripening and flower color formation [25]. The accumulation of phytoene is a concentration-limiting step in carotenogenesis, and PSY is commonly considered the prominent regulatory enzyme in this pathway. In this study, two orthologs of *PSY*, *Cla009122* and *Cla003169*, were found to be differentially expressed during fruit ripening in LSW177, whereas only *Cla009122* was found to be differentially expressed during fruit ripening in COS. The expression level of *Cla009122* in the two cultivars was low at ten DAP but rapidly peaked at 34 DAP in both cultivars, and the level in the red-flesh LSW177 was significantly higher than that in COS from 18 to 42 DAP during fruit ripening. This gene is upregulated in different red-flesh watermelon accessions, and its expression is significantly higher in these than in non-red-flesh watermelon during fruit ripening [1, 5, 7, 26]. In yellow-flesh tomato fruits, abnormal transcripts of *PSY1* and the loss of function of the enzyme result in a significantly reduced level of phytoene and a very low level of colored carotenoids. The *PSY* transcript abundance has been associated with improved carotenoid instability in the roots of maize [27]. It has been suggested that *Cla009122* is the *ClaPSY1* that is mainly responsible for carotenoid synthesis in watermelon fruit. Although PDS might play a concentration-limiting role in the generation of 9,15,90-tri-cis-ζ-carotene [28] and the gene expression levels of *ZDS*, *LCY*, *IPI*, *GGPS* and *PSY* are affected in the *pds3* mutant of *Arabidopsis thaliana* [29], we did not find any differentially expressed *PDS*-homologous genes between the two cultivars or during fruit development and ripening. ZDS and Z-ISO play important regulatory roles in the catalysis of ζ-carotene, the product of *PDS*, to tetra-cis-lycopene, the substrate for CRTISO. In this study, *ZDS* and *Z-ISO* were found to be differentially expressed between COS and LSW177 and were upregulated during fruit ripening in LSW177. In contrast, the expression of these genes in COS was nearly unchanged during fruit ripening and significantly lower than that in LSW177 from 26 to 42 DAP. The cyclization of lycopene is a key node of this pathway that produces β-carotene and α-carotene from lycopene by LCYB and LCYE, respectively [7, 30]. In this study, these two genes were found to be expressed similarly in the two cultivars and during fruit ripening, and the same results were found by RNA-Seq for 97103 and Dumara [1, 5]. Two previous quantitative studies used real-time PCR to study the *LCYB* expression level in different watermelon varieties. One study showed that low transcript levels of *LCYB* caused lycopene to accumulate in ‘CN62’ with pink flesh, and no significant differences in *LCYB* expression were detected between red-flesh “CN66” and yellow-flesh “ZXG381” [26]. Another study found no differential expression of *LCYB* between red (“Black Diamond” and “Festival Hybrid”)- and canary-yellow (“Yellow Doll” and “Yellow Sunshine”)-fleshed watermelon cultivars [31]. The continuously low expression level of *LCYB* and *LCYE* from the early to the mature stage was likely the main reason that the newly biosynthesized lycopene in LSW177 could not be further catalyzed to synthesize β-carotene or α-carotene and was gradually deposited in the vacuole to form red flesh. In our previous research, the red-flesh trait in the F2 and F3 population from COS × LSW177 was located in LG4; interestingly, *LCYB* was located between the two cleaved amplified polymorphic sequence (CAPS) markers WII04E07-33 and WII04E07-40, which were tightly linked to the red-flesh trait, with R^2^ = 83.5% [8]. In the *LCYB*-coding/promoter regions, a very small amount of activity of the LCYB enzyme induced by SNPs or INDEL likely results in lycopene accumulation [31–33]. Furthermore, we performed a selective sweep of the *LCYB* gene sequence of 20 watermelon accessions (Additional file 9) collected from the whole-genome sequence of watermelon [6], and the values of Pi, θ, and Tajima’s D in a population of wild and semi-wild watermelon were 0.0044, 0.0039, and 0.6273, respectively, whereas these values in a population of cultivated watermelon were significantly decreased to 0.0004, 0.0007, and -1.5622, respectively, suggesting that the genetic diversity of the *LCYB* gene decreased during watermelon evolution. These results suggest that *LCYB* might be a genetic determinant for lycopene accumulation in watermelon fruit. The *CHYB* and *ZEP* genes are involved in violaxanthin formation and lutein accumulation, respectively, and two orthologs of *CHYB* were found to be upregulated during fruit ripening in both cultivars. The same pattern was found in Dumara [7], 97,103 [1], CN66, CN62, ZXG381, and ZXG507. The expression of two *CHYB* genes in COS was higher than that in LSW177 during fruit ripening, whereas the expression in LSW177 was significantly higher than that in COS at the over-ripening stage. However, the expression of the two genes was markedly lower in the fruit mesocarp of cultivated watermelon and in the fruit flesh of wild watermelon [1]. *ZEP* expression was upregulated only in LSW177 but was lower and stable during fruit ripening in COS. In the yellow-flesh cultivated watermelon “ZXG381”, the transcript variations in *ZEP* and *CHYB* strongly correlated with changes in the violaxanthin and lutein contents during ripening [26]. In squash, the upregulation of *ZEP* and *CHYB* transcription levels leads to violaxanthin and lutein production [34], respectively. These results suggested that the expression level of *CHYB* and *ZEP* might help maintain sustainable carotenes levels in COS. A further analysis and exploration of carotenes in COS is required to determine their significant correlation in COS. *NCED* is also involved in the catabolic pathway, which converts 9-cis-violaxanthin or 9-cis-neoxanthin to xanthoxin, an ancestor of ABA that is important for non-climatic fruit ripening [23, 35, 36]. *NCED* genes were found to be differentially expressed during fruit ripening in both cultivars; *NCED2* showed mixed expression patterns, and *NCED4* expression was downregulated during fruit development. These genes did not present differences in expression between the two cultivars. The expression of only *NCED3* was upregulated in both cultivars during fruit growth, and the expression level of *NCED3* in COS was markedly lower than that in the corresponding LSW177 flesh tissues at the late stage of fruit ripening. In addition, the expression of *PSY1*, *Z-ISO*, and *CRTISO* is directly regulated by the ripening inhibitor (RIN) protein, which is a main member of the MADS-box family of TFs [37, 38]. In this study, three and five MADS-box TFs in COS and LSW177 were differentially expressed, respectively. One gene, *Cla000691*, is associated with *LeRIN-MADS* in tomato. The identification of watermelon *LeRIN-MADS* homolog genes, which present expression peaks throughout the maturation process in both cultivars, strongly supports this hypothesis. An additional member of the AP2/ERF superfamily, SlERF6, was recently found to play an essential role in tomato fruit ripening and carotenoid accumulation by acting as a negative regulator of two predominant nutritional compounds of tomato (lycopene and β-carotene). One watermelon gene (*Cla021765*) is similar to *SlERF6* and is downregulated during fruit ripening in both cultivars. Our comprehensive comparative expression analysis of genes involved in the carotenoid biosynthesis pathway between COS and LSW177 suggests that carotenoid accumulation and consumption in cultivated watermelon are significantly correlated with the key genes and TFs of this pathway.

Plant hormones play major roles in fruit development and (maturation) ripening [19]. In this study, 16 genes were found to be involved in plant hormone metabolism: 13 genes in ABA formation and signal transduction, and three genes in ethylene metabolism. Many studies have investigated the molecular mechanisms of fruit ripening in climacteric fruits. The investigation of maturation-defect mutant tomatoes has provided a significant amount of information, and ethylene has been identified as a significant phytohormone in climacteric fruits [39, 40]. Most studies of the ripening mechanism have been performed in non-climacteric fruits instead of climacteric fruits. Watermelon, a non-climacteric fruit, also produces ethylene but only at a trace level, although ABA accumulation occurs rapidly during ripening, which indicates that ABA might be involved in the regulation of watermelon maturation and senescence [41]. ABA synthesis and degradation regulate the ABA level in plants. ABA biosynthesis occurs downstream of the carotenoid biosynthesis pathway. NCED and ABA2 are the key concentration-limiting steps in ABA biosynthesis, and the ABA 8’-hydroxylase gene is essential for the catabolism of ABA [42]. In this study, the upregulated expression of *NCED2* genes in COS and LSW177 was found to aid the shift from carotenoid metabolism to ABA metabolism during fruit development depending on the increase in ABA content during watermelon fruit growth and ripening, which as previously mentioned [41], might be the main driver of cultivated watermelon maturation. The *NCED* gene also plays a primary function in the biosynthesis of ABA during fruit maturation [19, 36] in tomato [43] and melon [44]. Thus, NCED might be a noteworthy regulator of the ripening phase in cultivated watermelon. The expression of an ortholog of *ABA 8’-hydroxylase*, *Cla005457*, was relatively low at the early stage, increased rapidly with fruit ripening, and peaked at 26 DAP in both cultivars, and its expression in COS was higher than that in LSW177 during fruit ripening. These results suggest that the internal ABA content differs between the two cultivars. The biosynthesis and catabolism of ABA are regulated by *BG* genes during ripening and development [41]. In this study, eight orthologs of *BG* were found to be differentially expressed during ripening in both cultivars; two of them were upregulated, and the others were downregulated or showed a biphasic expression pattern in both cultivars. A previous study showed that *ClBGs* with differential expression patterns in watermelon appear to have overlapping functions in ABA catabolism during watermelon ripening [41]. However, the expression of *BG* remained at its peak from the coloration step to fruit ripening to regulate the levels of ABA during melon and grape ripening [44, 45], which indicates that *BG* has an important complex function during watermelon ripening. However, the precise contribution of *BG* to the ABA levels in watermelon fruit requires further study. For ABA signal transduction, the preliminary reaction to ABA implies the ABA-dependent PYR/PYL-mediated inactivation of PP2Cs, which permits the release of SnRK2s and the phosphorylation of ABA-dependent TFs. In our study, *PYL/PYL*, *PP2Cs* and *SnRK2s* showed differential expression patterns between COS and LSW177 during fruit ripening. Therefore, the findings of this study indicate that different transcriptional regulatory mechanisms modulate ABA reactions during different watermelon fruit ripening stages. Ethylene plays a primary role in the ripening of climacteric and non-climacteric watermelon fruits. *ACS*, *ETR* and *ERF* are key genes in the ethylene formation and transduction pathway and were found to be differentially expressed during fruit ripening in COS and/or LSW177. No ACS gene was differentially expressed in the flesh of LSW177 during watermelon ripening, whereas an ortholog of *ACS* in COS was differentially expressed, although at extremely low levels, during fruit ripening. The expression of *ACS*-homologous genes in watermelon is also low [1], and previous studies have found that the ethylene synthesis rate in watermelon is markedly lower than that in tomato [3], which might at least partly explain the differential expression of the *ACS* gene. Ethylene is identified by the receptors (ETR) and additional related proteins, and ETR functions as a negative regulator of ethylene reactions in tomato [46, 47]. In this study, an *ETR*-homologous gene was found to be consistently downregulated in both cultivars and presented a higher expression level in COS compared with that in LSW177 at the early phase of fruit ripening. A similar expression trend for an ETR-homologous gene was found in the fruit flesh of 97103 [1]. Moreover, we used the numerous TFs that were differentially expressed to normalize the behavior of ethylene-biosynthesis-related genes, such as the TFs of the AP2-ERFBP and MADS-MIKC superfamily. The TFs of the AP2-EREBP family have either one or two AP2 domains, the name of which derives from APETALA2 protein [48]. In tomato, APETALA2 (SlAP2a) influences fruit maturation by regulating ethylene formation and signaling [46]. SIAP2a is synthesized at low levels in flowers and early phases of fruit ripening but is considerably upregulated from the mature green to the breaker stages and is noticeably synthesized during the red-ripe stage. A total of 38 and 31 *AP2-ERFBPs* in COS and LSW177, respectively, were found to be differentially expressed. Of these, *Cla000701* was significantly upregulated during fruit ripening in both cultivars, and it has been suggested that this TF is an important functional ortholog of SlAP2a in watermelon, although its role in non-climacteric ripening might be different from that in ethylene regulation.

Sugars are crucial components of cultivated watermelon fruit quality [49] and serve as imperative signals in the regulation of fruit ripening [19, 50]. As with all other fruits of cucurbits, watermelon sugars consist of sucrose, fructose and glucose [51]. The sweetness level of watermelon is determined by calculating the total sugar contents and by determining the composition of glucose, fructose and sucrose [52]. At the early stage of ripening, the sucrose content in watermelon is very low but increases at later stages, whereas the fructose and glucose contents remain constant [49, 51]. In this study, the TSS, fructose and sucrose contents were found to be very low at the early stage of fruit development and increased rapidly at later stages in COS and LSW177, whereas the glucose content peaked in the early stage and then decreased to its initial level during fruit ripening. Dumara and 97,103 exhibited the sugar compositions and the same dynamic changes in the sugar content during fruit ripening [1, 5, 7]. The sugar content in watermelon fruit is evaluated by measuring the activities of phloem unloading and metabolism that occur within fruit flesh. Stachyose, raffinose and sucrose are the main sugars transported from the leaves to the fruit in the phloem of cucurbit plants [53]. Furthermore, a considerable amount of translocated sugars undergoes phloem loading, distribution and metabolism, which are controlled by pivotal sugar metabolism enzymes [49]. In this study, more differentially expressed genes were significantly enriched in pathways related to the biosynthesis/catabolism of stachyose, raffinose and sucrose, such as ‘galactose metabolism’, ‘pentose and glucoronate interconversions’, ‘other glycan degradation’, ‘fructose and mannose metabolism’, ‘glycolysis/gluconeogenesis’, and ‘starch and sucrose metabolism’. Several important genes encoding key sugar metabolism enzymes that are involved in these complex metabolic pathways were differentially expressed between COS and LSW177, and these included genes encoding raffinose synthase, α-galactosidase, SuSy, SPS, IAI, and UDP-sugar pyrophosphorylase (USP). In addition, these genes were also differentially expressed at different ripening phases in COS and LSW177. The raffinose synthase gene is critical for the synthesis of raffinose from galactinol. Six raffinose gene orthologs were found to be differentially expressed during fruit ripening in COS and/or LSW177; of these, *Cla017113* was consistently and equally upregulated in both cultivars, and its expression level in COS was higher than that in LSW177. The other raffinose gene orthologs were downregulated or showed biphasic expression patterns in COS and LSW177. α-Galactosidase is the core enzyme that hydrolyzes stachyose and raffinose and determines the sink strength in all cucurbit plants. An α-galactosidase-homologous gene, *Cla007286*, was found to be upregulated during fruit ripening, and its expression peak in COS was higher than that in LSW177. These domino effects indicated that *Cla007286* might function as an essential element for phloem unloading and sink strength evaluation during development. Previous studies have suggested that the sugars in watermelon are inspected primarily by three enzyme families: SuSys, SPSs and invertases [49, 51]. SuSy is a focal enzyme that can catalyze both the formation and hydrolysis of sucrose in plants. A positive correlation between SuSy activity and fruit sucrose accumulation has also been found in melon [54] and watermelon [51]. In this study, the gene expression level of one *SuSy*-homologous gene (*Cla011131*) was found to be upregulated during fruit ripening and presented a noticeable positive correlation with the sucrose content in LSW177, which suggests that this enzyme plays a vital role in evaluating the sugar composition in LSW177. SPS is a pivotal enzyme that catalyzes sucrose formation. SPS movement is positively related to sucrose accumulation in tomato [55], melon [56] and watermelon [51]. One ortholog of *SPS*, *Cla010566*, was found to be significantly upregulated in COS during fruit ripening, which suggests that the synthesis and accumulation of sucrose in COS and LSW177are regulated by different genes or mechanisms. In watermelon, sucrose translocation occurs equally with phloem unloading, is related to fruit sinks and requires insoluble acid invertase. Three *IAI*-homologous genes were found to be significantly differentially expressed and were consistently downregulated during fruit ripening in COS and/or LSW177, which suggests that the IAI activities are negatively correlated with sucrose accumulation during development in COS and LSW177. A noteworthy positive correlation between invertase activity and fruit sucrose accumulation has been reported in sweet watermelon [51], melon [57], tomato [58], and sugar cane [59]. In the proposed pathway for galactose metabolism, UPS exerts a positive effect on melon fruit sink metabolism [60], and an ortholog of *UPS*, *Cla013902*, was found to be steadily upregulated over time in the flesh of the investigated cultivars and exhibited a higher expression level in COS than in LSW177 during watermelon fruit development. A gene that exhibited the same trend was identified in 97103, but its expression remained relatively constant and was lower in the flesh of PI296341-FR and 97103 mesocarp, which indicates that the *UPS* gene functions in fruit sugar metabolism in watermelon. Two core catalyst superfamily sugar transporters were significantly and equally upregulated in the flesh of the investigated cultivars and had higher expression levels in COS than in LSW177 during watermelon fruit development. In contrast, their expression in the mesocarp of 97103 was greatly inferior to that in the flesh of 97103 and was almost absent in PI296341-FR flesh tissue. Moreover, these transporters were positioned at the flanking region associated with fruit sugar substance and major QTL *Qbrix2-2* on watermelon chromosome 2. In strawberry, the RNAi of the sugar transporter could significantly decrease the sucrose content and over-ripening of fruit [50]. Thus, the results reveal that transporter genes might participate in maximizing the sugar content in fruit flesh by providing the active transmembrane transport of sugars.

Cell wall metabolism is one of the most important variables in fruit ripening and is related to flesh texture [43], which is a significant quality attribute because it is directly associated with fruit commercial quality, including mouth feel, fruit durability, transportation and shelf life [1]. The fruit cell wall contains mainly pectin, cellulose, and hemicellulose, which form interlaced networks with diverse families of cell-wall-modifying proteins. The degradation of pectin and cellulose depends on multiple plant hormones that affect cell wall catabolism during fruit softening. Recent studies have revealed that fruit softening is regulated by a subset of major metabolism genes that contribute to cell wall metabolism during fruit ripening [1, 43], including *GAUT*, *PG*, *PE*, *BG*, *pectinesterase inhibitor* (*PEI*), *polygalacturonase inhibitor* (*PGI*), and *α-mannosidase* (*MANA*) genes. The *GAUT* gene is key for the synthesis of pectin [61], which is the core constituent of the primary cell wall and determines fruit consistency and quality. Pectin depolymerization is the main reason for decreased fruit firmness. The enzymes responsible for pectin variation in the fruit cell wall are PE and PG. PE catalyzes the hydrolytic de-esterification of pectin, resulting in pectin chain esterification, and the products are hydrolyzed to pectate by PG to result in tissue softening during ripening. The comparison of COS with LSW177 revealed that the *PG* and *GAUT* genes were upregulated in COS, whereas the *PE* genes displayed mixed expression patterns. In addition, the action of pectinesterase and polygalacturonase can be synchronized by protein inhibitors [62]; in this study, an *PEI* and an *PGI* were found to be significantly downregulated during fruit ripening in both cultivars, whereas COS had a lower *PEI* expression level and a higher *PGI* expression level compared with LSW177 at the early phase of ripening. The increasing activity of α-mannosidase has been significantly correlated with fruit softening and ripening during fruit ripening in mango [63] and tomato [64]. In this study, an ortholog of *MANA* was found to be significantly upregulated in both cultivars and presented a higher expression level in COS than in LSW177. Cellulose is another essential constituent of plant cell walls that provides mechanical support to the plant structure [65]. Endoglucanase is a key enzyme that catabolizes cellulose in plants. In this study, an ortholog of endoglucanase was found to be significantly upregulated from 18 to 42 DAP during fruit ripening, and its expression level was higher in COS than in LSW177. The above-described results suggest that the genes involved in cell wall metabolism might play critical roles in determining fruit texture, and most of these genes were differentially expressed between red-flesh and yellow-flesh watermelon fruits during ripening.

## Conclusions

Due to its non-climacteric behavior, watermelon is a model fruit, although the genes associated with fruit development and ripening remain largely unknown. A comparative transcriptome analysis of two contrasting watermelon genotypes during fruit development and ripening would provide additional information regarding the genetic basis of variations in fruit development. Using high-throughput RNA-Seq, we generated approximately 859 million high-quality reads from red-flesh and pale-yellow-flesh cultivated watermelon at the most important stages of fruit development. This dataset provides an accurate transcriptional status of the watermelon growth phase and provides the first gene expression profiles of a pale-yellow-flesh watermelon during development. An investigation of the gene expression profiles noted that many processes associated with watermelon fruit quality (such as sugar content, flesh color, texture) are regulated at the transcriptional level to a great extent. A number of key genes were identified by evaluating watermelon fruit characteristics together with sugar metabolism and transport, carotenoid biosynthesis, and cell wall metabolism. Hence, our investigation provides a method for guiding the detection of fundamental genes. Regulatory genes with well-ripening-associated functions, such as those involved in the ABA, ethylene biosynthesis and signaling pathways, were also identified. The expression patterns of these genes suggest that ABA and ethylene might equally contribute to regulating watermelon fruit quality, although watermelon is categorized as a non-climacteric fruit. Our comparative transcriptome study provides new genome-wide insights into the molecular-level mechanisms of fruit development and ripening and the regulation of numerous essential fruit quality attributes of watermelon, such as sugar accumulation, flesh color and flesh texture.

## Methods

### Plant cultivation

The watermelon [*Citrullus lanatus* (Thunb.) Matsum. & Nakai var. lanatus] cultivars LSW177 and COS were used in this study. LSW177 is a famous global commercial cultivar that bears elongated fruits at the mature stage with a firm, crisp, red flesh (rich in lycopene) and with a low total soluble sugar content for consumers who prefer fruits with low sugar and carbohydrate contents [66]. In contrast, COS (Cream of Saskatchewan) has spherical fruits characterized by a green rind, dark green stripes and very pale yellow flesh (low carotenoid contents). This cultivar is important for flesh color studies [24, 67]. Seedlings were grown in pots (filled with nutritional media) in a greenhouse on 20 May 2014, and one-month-old watermelon seedlings were transplanted into rectangle rows with fertile soil in a greenhouse on the Xiangfang Farm of Northeastern Agriculture University (at approximately 44.04 N and 125.42 E). After transplanting, the plants were separated by genotype and replication and placed under field management using standard horticultural procedures, such as irrigation, hand weeding, and pathogen prevention and control, with were implemented according to the methods used by local farmers based on the climate of Harbin City (about 12-h day length; 25 °C average temperature in summer). In addition, flowers were hand-pollinated and tagged to record the number of days after pollination (DAP). Flesh samples for RNA extraction were collected randomly from the center of five injury-free watermelon fruits from every cultivar at five different ripening stages (10, 18, 26, 34, and 42 DAP; Fig. 1). These samples were immediately frozen in liquid nitrogen, delivered rapidly to the laboratory and stored at -80 °C until use.

### Measurement of the soluble sugar and lycopene contents

Approximately 5 g of flesh was used to analyze the lycopene content, as previously published [8]. Every sample was examined using three replicates. A UV spectrophotometer was used to analyze the soluble sugars (total soluble solid, glucose, fructose, and sucrose) as previously described [68].

### RNA-Seq library preparation and sequencing

The total RNA from frozen watermelon fruit flesh from every fruit stage was isolated using the RNAplant Plus Reagent Kit (TIANGEN, Beijing, China) according to the manufacturer’s instructions. The quality, quantity, and integrity of the total RNA were evaluated using a NanoPhotometer® spectrophotometer (IMPLEN, CA, USA), the Qubit® RNA Assay Kit with a Qubit® 2.0 Fluorometer (Life Technologies, CA, USA) and the RNA Nano 6000 Assay Kit with a Bioanalyzer 2100 system (Agilent Technologies, CA, USA), respectively. Briefly, 6 μg of RNA per sample was used as input material for preparation of the RNA samples. The total RNA samples were treated with RNase-free DNase I and then subjected to poly-A RNA enrichment using poly-T oligo-attached magnetic beads. First-strand cDNA was synthesized using a random hexamer primer and M-MuLV Reverse Transcriptase (RNase H). Second-strand cDNA synthesis was subsequently performed using DNA polymerase I and RNase H. After cDNA library construction, clusters were generated according to the manufacturer’s instructions. The library preparations were sequenced with an Illumina HiSeq™ 2500 at the Novogene Bioinformatics Institute in Tianjin, China. High-quality reads (clean reads) that were 125 bp in length were obtained by deleting low-quality reads with vague nucleotides and filtering adapted sequences from the crude reads.

### Transcript assembly and DEGs analysis

We used the watermelon reference genome assembly (v1) reported by Guo et al. [6], and extension was performed using the available gene predictions with transcript sequence evidence. To this end, after trimming and quality control, the available reads of high quality were aligned to the referred genome using a genome-guided assembly approach with TopHat (v2.0.10) and the default parameters [9], and a wide-range transcriptome containing the RNA sequencing data of a total of 10 dissimilar tissues and cultivars was built and merged using Cufflinks (v2.2.1) as previously described [9]. We then compared the transcripts from our study with the annotated transcripts of the reference genome using Cuffcompare of Cufflinks. The transcripts with the class codes ‘i’, ‘u’, and ‘x’ were selected as novel transcripts and were implemented as a verified update to the existing feature annotation of the reference transcriptome. To obtain a functional annotation, open reading frames (ORFs) were predicted from the novel transcripts using TransDecoder (v2.1.0) (http://transdecoder.github.io/) with default parameters. We then blasted these ORFs against the SwissProt database (Evalue = 1e-5), which contains a relatively small but informative set of proteins. HMMER (v3.1b2) was used to compare the model profiles from the Pfam database with default parameters [69]. To annotate the ORFs resulting from these sequence comparisons, we searched the ORF sequences from the protein databases, including the Nr [11], SwissProt [12], GO [13], and KEGG [14] databases, by BLASTX (Evalue = 1e-10). We also fetched the proteins analogous to the given ORF sequences along with their protein functional annotations. The Blast2GO (v3.0) program was used to determine the GO annotations of the ORFs with default parameters [70].

To identify the DEGs, we used a Python script, HTSeq-count (v0.6.1p2) [71], to calculate the clean read counts of each gene according to the merged transcripts created by Cuffmerge as described in Trapnell et al. [9]. Prior to variational expression analysis with the edgeR package (v3.10) [72], we examined the whole affinity between the samples to confirm that they met the desired expectation of the experimental plan. We performed a principal-component analysis (PCA) to compare the samples and thereby identify the components that best clarify the variance in the data, as shown in Additional file 5: Figure S3 (Additional file 1). The DEGs were then determined using the edgeR protocol as previously described [73], and the DEGs were defined as significant based on a false discovery rate (FDR) with a Benjamini and Hochberg [74] corrected *P* value ≤ 0.05 and an absolute value of log_2_Ratio ≥ 1. To increase power, we removed those genes with less than three counts in the overall samples, which decreases the load of a strong multiple-test correction [75]. Gene expression was calculated using well-mapped reads, and the results were normalized to the fragments per kilobase of exon per million mapped fragments (FPKM).

### Functional analysis of DEGs

The DEGs were analyzed for GO classifications using Blast2GO and WEGO [76], and their KEGG pathway annotation was analyzed by KOBAS (v2.0) (http://kobas.cbi.pku.edu.cn/). KOBAS detects statistically significantly enriched pathways using a hypergeometric test and has been effectively used for the differential pathway analysis of living entities, such as plants, animals and bacteria [77]. DEG clustering was performed by STEM using the default parameters [15]. This algorithm uses exclusive methods for clustering, comparing, and visualizing data and provides useful and statistically rigorous biological explanations of short time-series data due to its integration with GO. The GO enrichment of co-expressed DEGs was performed using the hypergeometric distribution algorithm. Clustered profiles with a *P* value ≤ 0.05 were considered differentially expressed.

### Selective sweep analysis on *LCYB*

The *LCYB* sequences of 20 watermelon accessions (cultivated, semi-wild and wild populations) were collected from previous research [6]. The genetic diversity of *LCYB* was analyzed using MEGA (v6.0) with default parameters as previously described [78]. Selective sweep analysis is usually used to identify genomic regions that have been targets of selection during domestication based on differences in the genetic diversity or DNA polymorphisms of a population, which are commonly estimated by Pi, θ, and Tajima’s D in selective sweep analysis [79, 80].

### TF analysis

To determine the most suitable TF families that play a pivotal role in the development and ripening of watermelon fruit, the DEGs in COS and LSW177 during fruit ripening were classified as putative TFs based on predictions using the PlantTFcat database [18]. The putative TFs within the DEGs were divided based on their TF families and their presence in COS and LSW177.

### Gene expression analysis by qPCR

Sixteen genes that are important for fruit ripening were selected to validate the RNA-Seq results by qPCR. The primers that were used for the amplification of these genes are provided in Additional file 10 and were designed with Primer Premier (v6.0) software [81]. For primer design, minute amplicons (90-200 bp) within the first third of the cDNA sequences were preferred. Whenever possible, forward and reverse primers were bound to different exons, and the reverse primer was designed to hybridize with two consecutive exons to avoid the amplification of genomic DNA.

A total of 1 μg of RNA was reverse transcribed for first-strand cDNA synthesis using the EasyScript® One-Step gDNA Removal and cDNA Synthesis SuperMix (TransGen Biotech, Beijing, China) according to the manufacturer’s instructions. A 20-μL reaction was prepared with 10 μL of SYBR Green Master mix (TOYOBO, Osaka, Japan), 1 μL of each primer pair and 1 μL of cDNA templates. The PCR amplification of the target genes was performed in 96-well optical reaction plates on an iQ5 Gradient Real Time PCR system (Bio-Rad, Berkeley, CA, USA). A total of two biological replicates and three technical replicates were used for each cultivar and ripening stage assayed. The thermal cycling program was as follows: 95 °C for 10 s; 40 cycles of 95 °C for 15 s, 60 °C for 30 s and 72 °C for 30 s; a final melt curve analysis in which the temperature was increased from 55 °C to 95 °C at a rate of 0.5 °C/5 s; and a maintenance at 4 °C. The specificity was verified by melt curve analysis and agarose gel electrophoresis of the amplified products. The relative quantification of gene expression levels was performed using the 2^-ΔΔCT^ method [82]. The *Cla020175* gene (yellow leaf specific protein 8, *CIYLS8*) was used for normalization of the assayed genes because its expression does not vary in watermelon organs and tissues under typical growth conditions or in the presence of abiotic stress or biotic stress [83]. The mean fold-change values were used for graphical representation.

## Additional files

Additional file 1: Data (zip file) of the evidence-based novel genes identified in this study. (ZIP 250 kb)
Additional file 2: Excel file containing the annotations of the novel genes. (XLSX 178 kb)
Additional file 3: Excel file containing the DEGs in COS at five developmental stages. (XLSX 562 kb)
Additional file 4: Excel file containing the DEGs in LSW177 at five developmental stages. (XLSX 491 kb)
Additional file 5: Figure S1. qPCR validation of differential expression. The transcript levels of 16 genes, including 13 involved in carotenoid biosynthesis (A ~ M) and three TFs most likely associated with fruit development and ripening (N ~ P), in COS (broken lines) and LSW177 (solid lines) and the corresponding RNA-Seq expression data. COS-F: the data for RNA-Seq in COS; LSW177-F: the data for RNA-Seq in LSW177. The principal y-axis shows the relative gene expression levels analyzed by qPCR, and the secondary y-axis shows the RNA-Seq data. The bars represent SE (*n* = 3). (Q) Comparison of the gene expression ratios obtained from RNA-Seq and qPCR. The RNA-Seq log2 value of the expression ratio (y-axis) was plotted against the developmental stages (x-axis). **Figure S2**. GO classification of the upregulated DEGs (A) and downregulated DEGs (B) in COS and LSW177. X: function items. Y (left): gene percent. Y (right): number of genes. Blank items (A): genes upregulated in LSW177. Gray items (A): genes upregulated in COS. Blank items (B): genes downregulated in LSW177. Gray items (B): genes downregulated in COS. **Figure S3**. PCA analysis to examine the similarity between samples. “C” (green) and “L” (blue) represent COS and LSW177, respectively. The numbers after “C” and “L” represent the number of days after pollination. (DOCX 903 kb)
Additional file 6: Excel file containing the KEGG pathway annotations for the DEGs. (XLSX 40 kb)
Additional file 7: Excel file containing the KEGG pathway annotations for 583 DEGs. (XLSX 16 kb)
Additional file 8: Excel file containing the TF annotations. (XLSX 33 kb)
Additional file 9: Summary information and sequence of the LCYB gene in 20 watermelon accessions. (DOCX 19 kb)
Additional file 10: Excel file containing the primer sequences used for real-time PCR. (XLSX 10 kb)

## Abbreviations

ABA: Abscisic acid
CAPS: Cleaved amplified polymorphic sequence
CHYB: β-carotene 3-hydroxylase
DAP: Days after pollination
DEGs: Differentially expressed genes
ESTs: Expressed sequence tags
FDR: False discovery rate
GGPP: Geranylgeranyl diphosphate
MANA: α-mannosidase
NCED: 9-cis-epoxycarotenoid dioxygenase
ORFs: Open reading frames
PCA: Principal component analysis
PEI: Pectinesterase inhibitor
PGI: Polygalacturonase inhibitor
PSY: Phytoene synthase
qPCR: Quantitative real-time PCR
STEM: Short time-series expression miner
TF: Transcription factor
TSS: Total soluble sugar
USP: UDP-sugar pyrophosphorylase
Z-ISO: ζ-carotene isomerase
