# Supplementary figures and images for: Comparative transcriptome analysis of two contrasting watermelon genotypes during fruit development and ripening
Source: BMC Genomics. 2017 Jan 3;18:3. doi: 10.1186/s12864-016-3442-3 (PMC5209866; doi:10.1186/s12864-016-3442-3)

Figure S1.


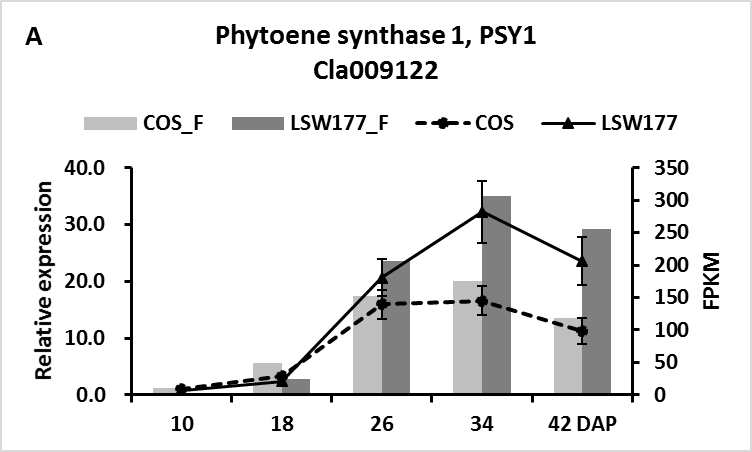

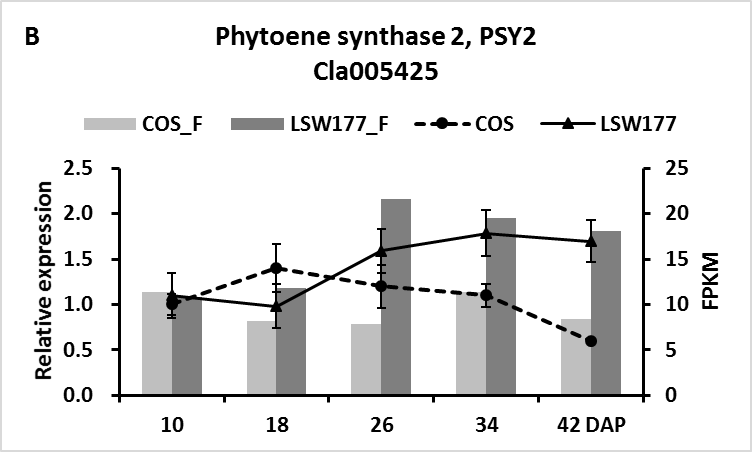


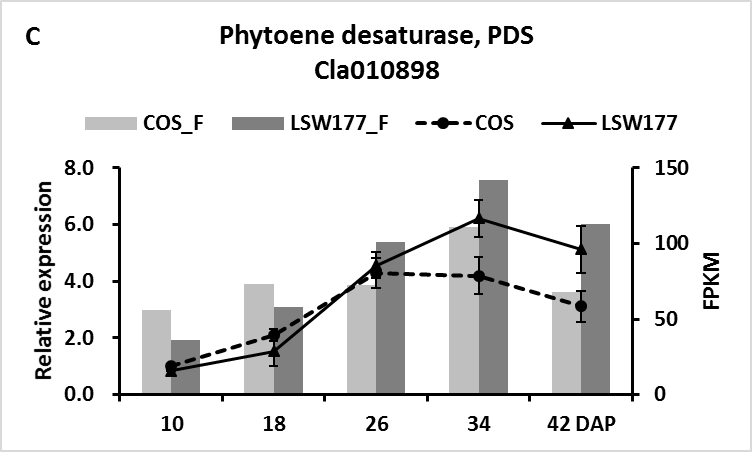

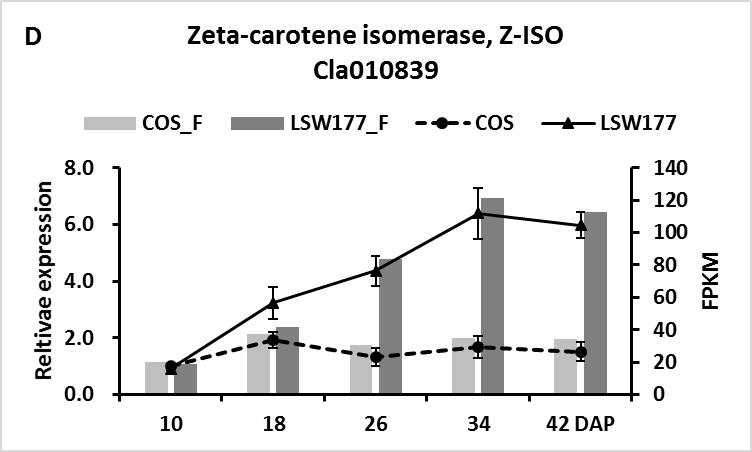


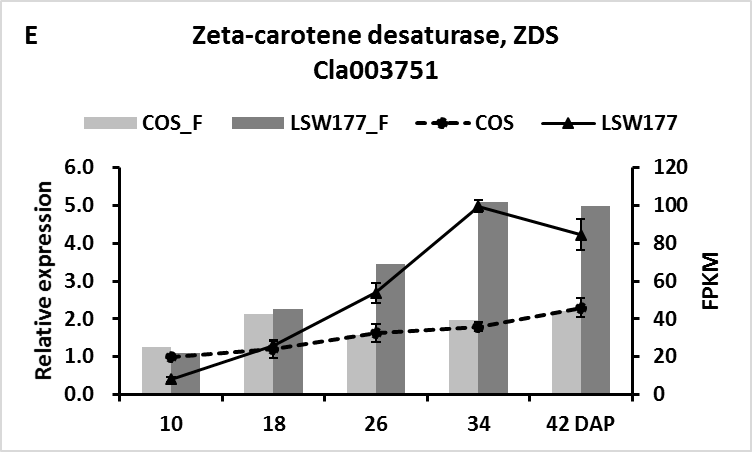

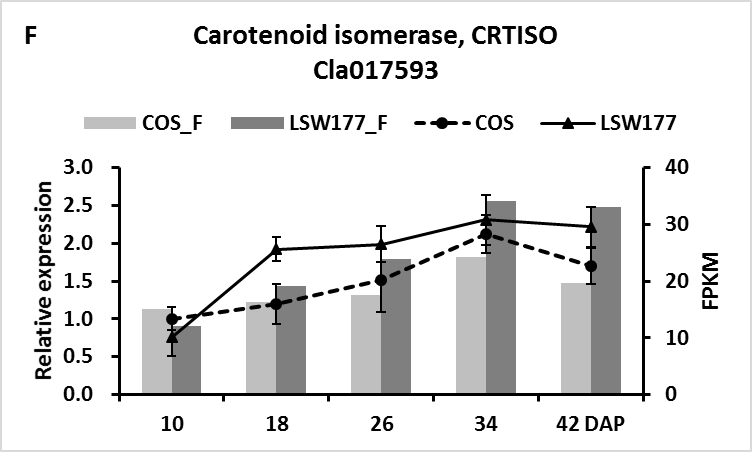


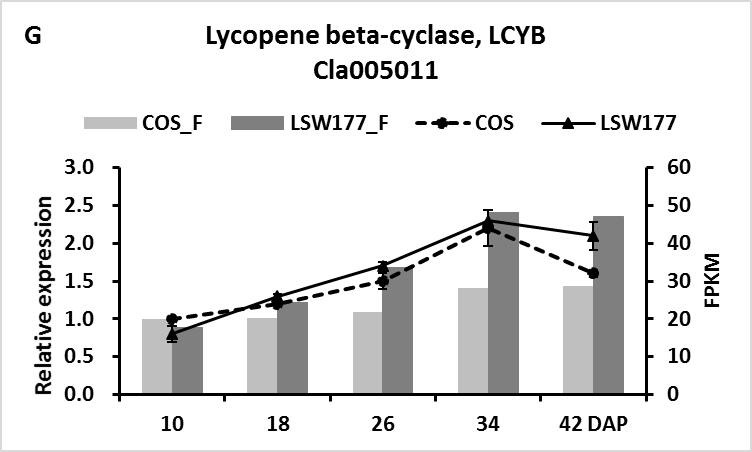

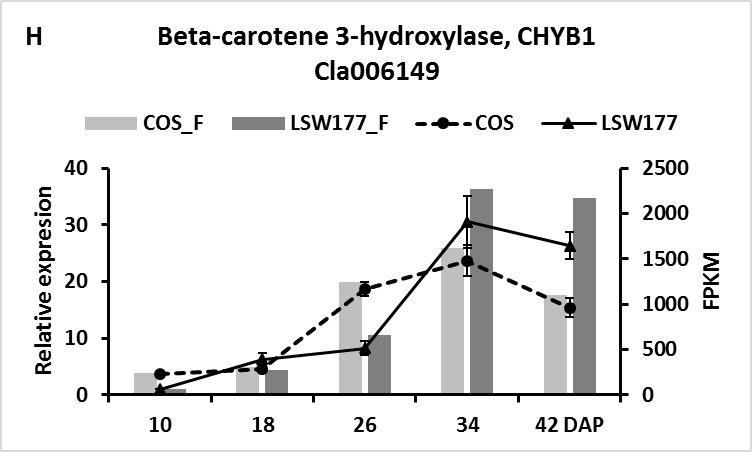


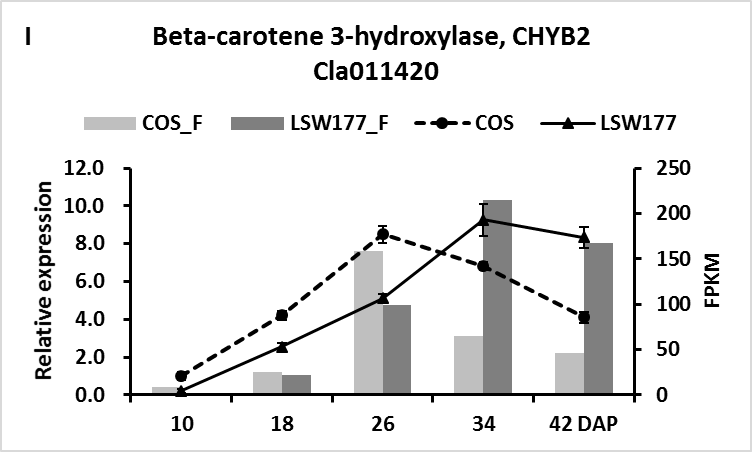

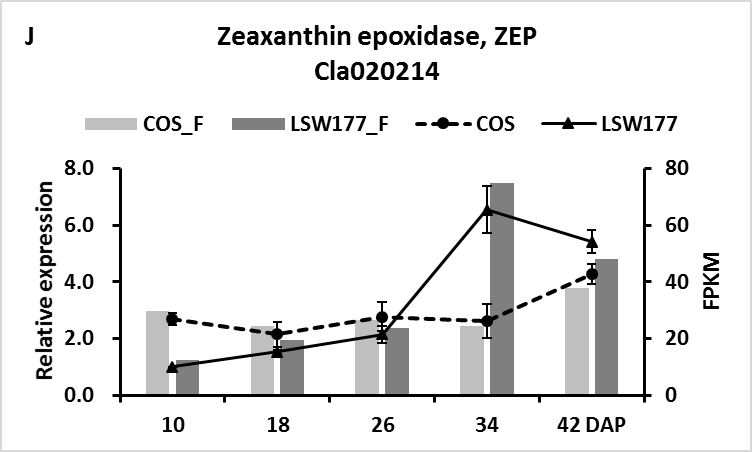


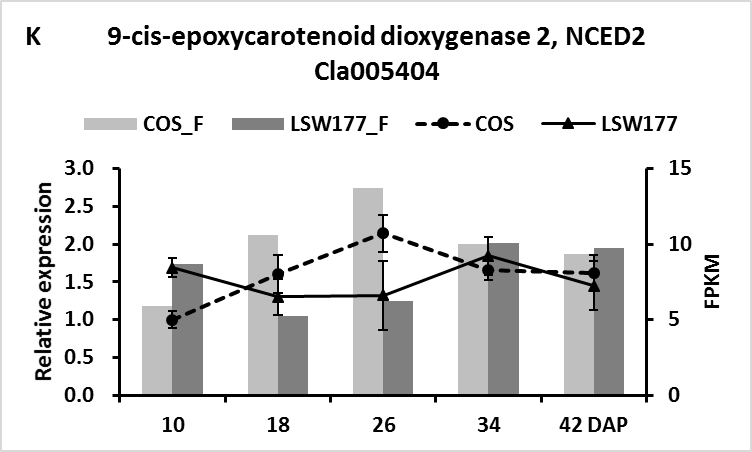

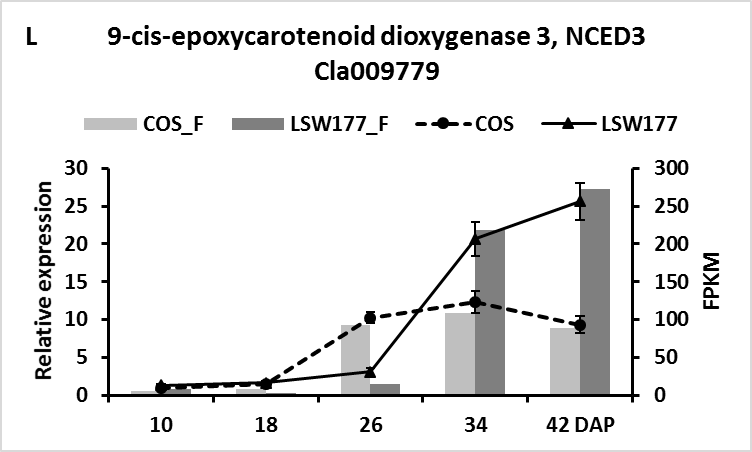


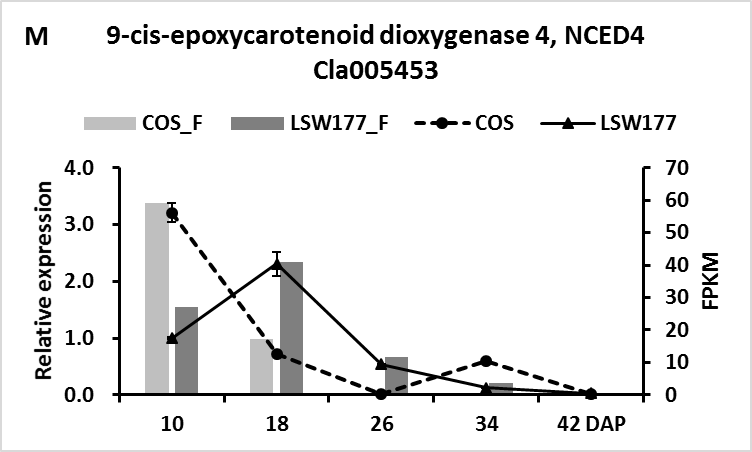

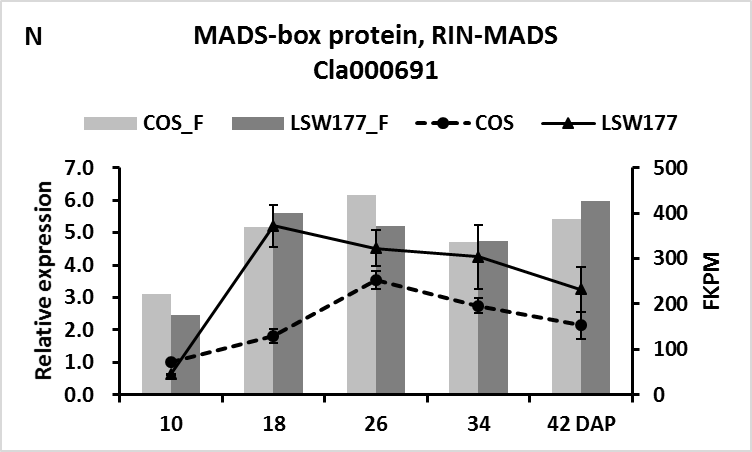


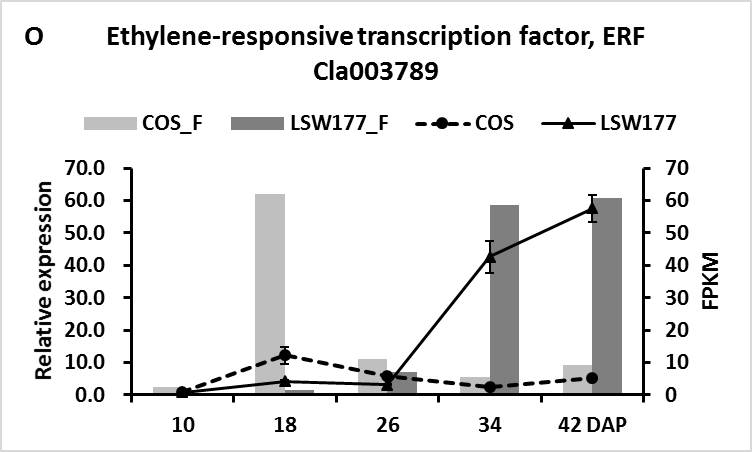

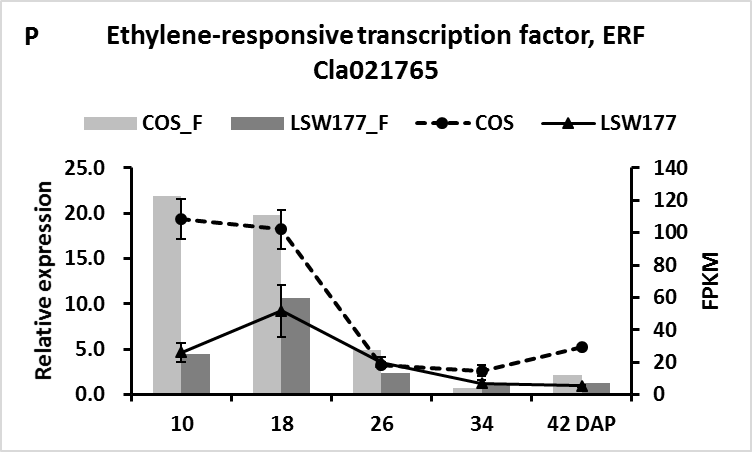


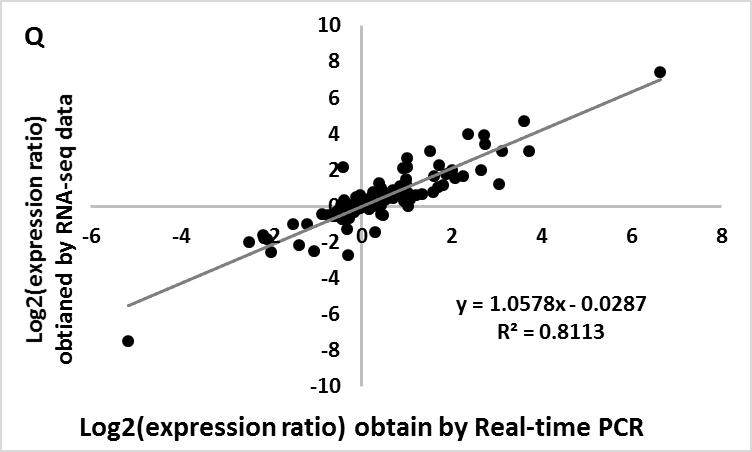


Figure S2.


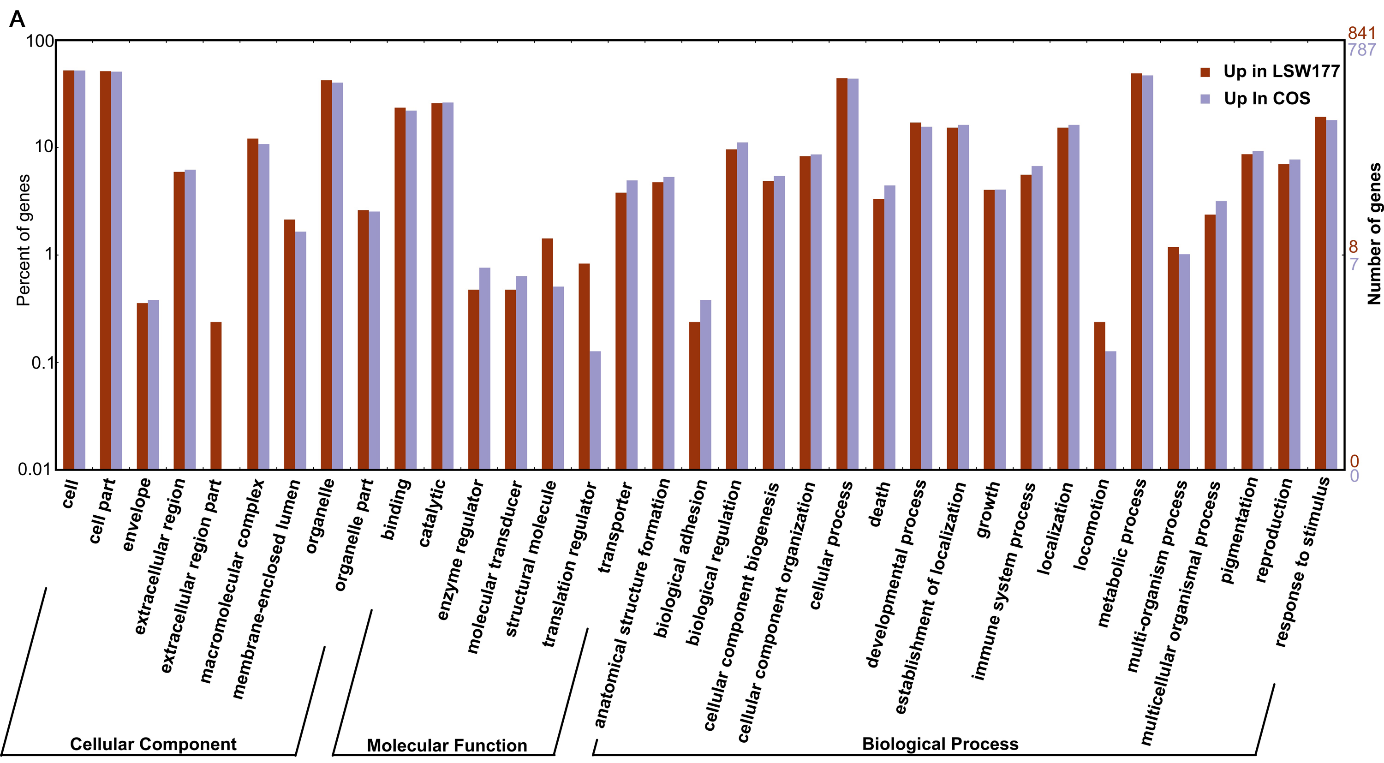


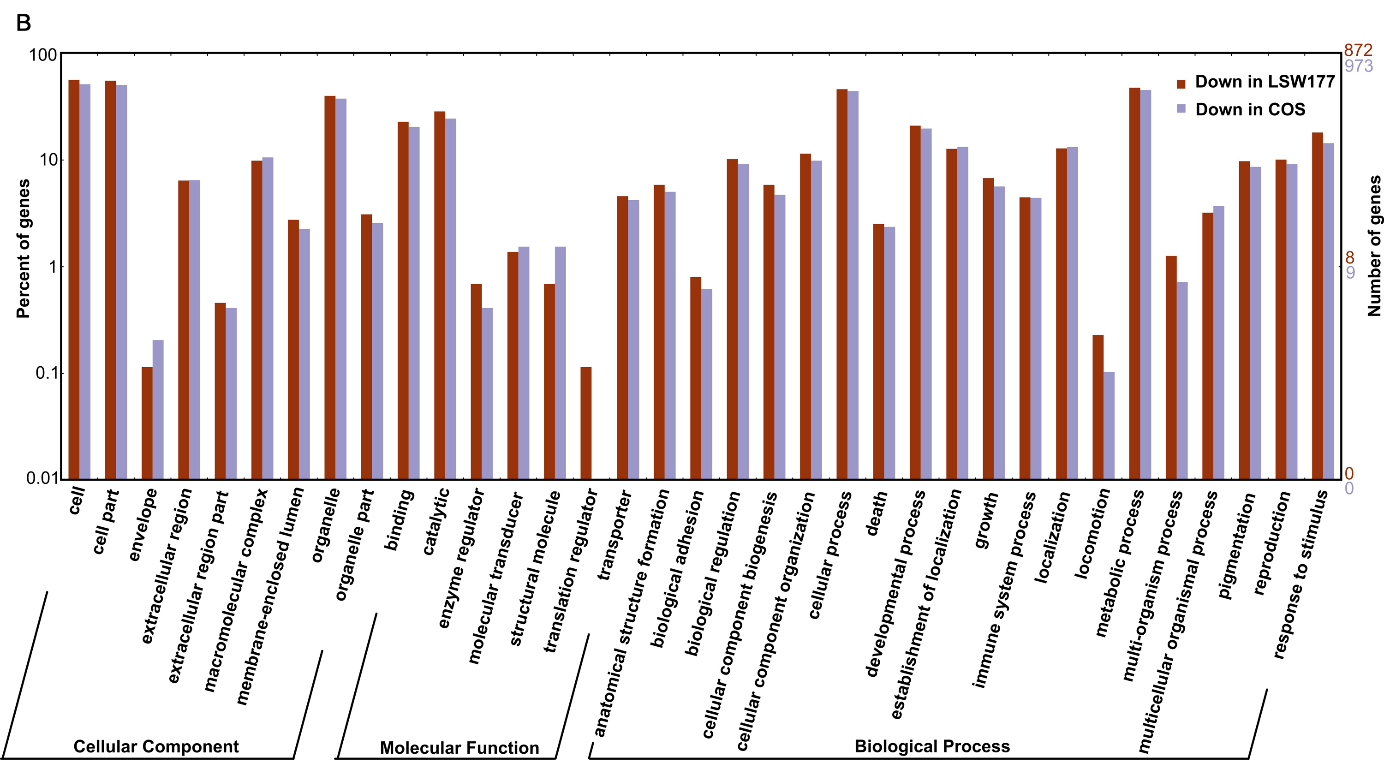


Figure S3.


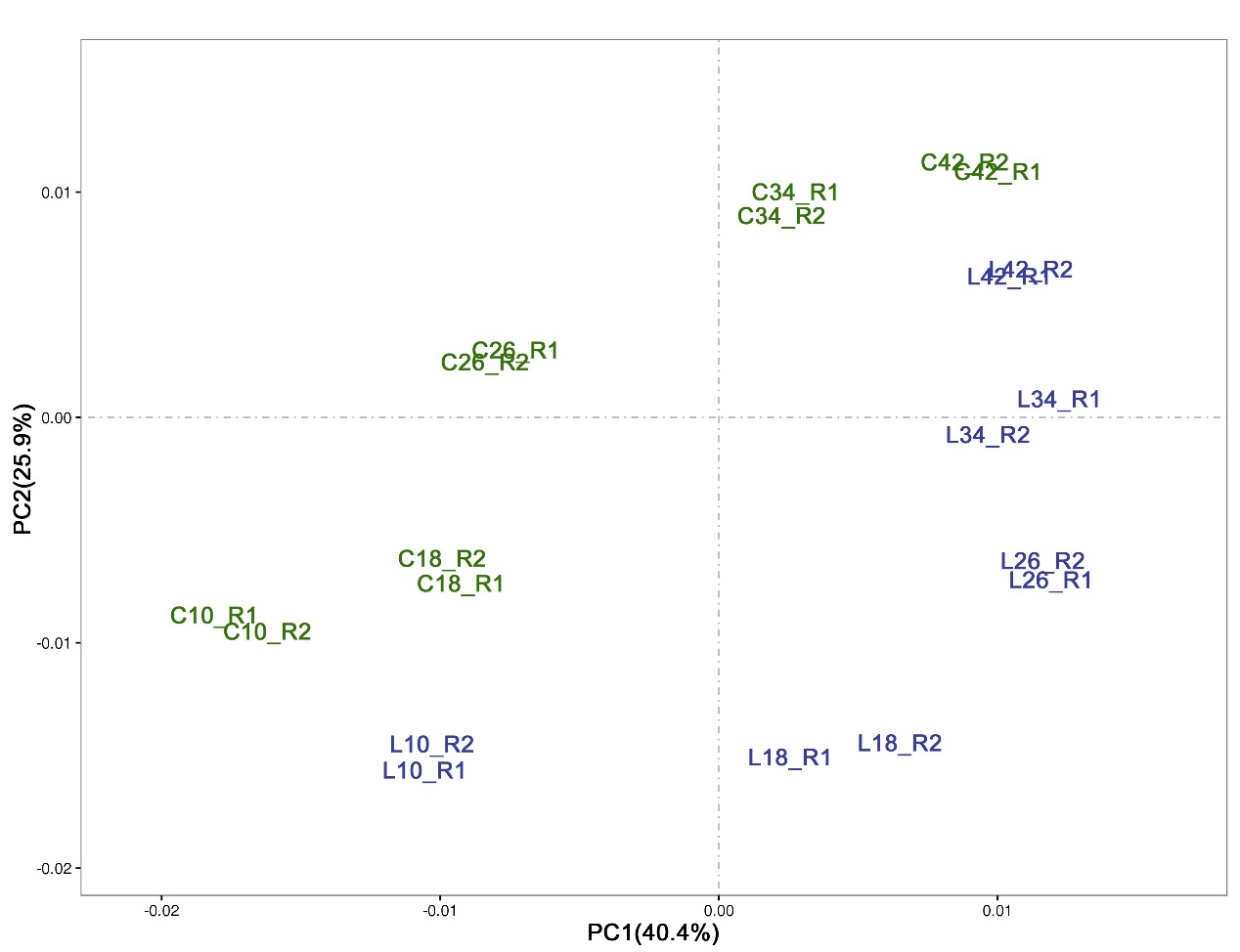

Supplement: Additional file 5: Figure S1. — qPCR validation of differential expression. The transcript levels of 16 genes, including 13 involved in carotenoid biosynthesis (A ~ M) and three TFs most likely associated with fruit development and ripening (N ~ P), in COS (broken lines) and LSW177 (solid lines) and the corresponding RNA-Seq expression data. COS-F: the data for RNA-Seq in COS; LSW177-F: the data for RNA-Seq in LSW177. The principal y-axis shows the relative gene expression levels analyzed by qPCR, and the secondary y-axis shows the RNA-Seq data. The bars represent SE (n = 3). (Q) Comparison of the gene expression ratios obtained from RNA-Seq and qPCR. The RNA-Seq log2 value of the expression ratio (y-axis) was plotted against the developmental stages (x-axis). Figure S2. GO classification of the upregulated DEGs (A) and downregulated DEGs (B) in COS and LSW177. X: function items. Y (left): gene percent. Y (right): number of genes. Blank items (A): genes upregulated in LSW177. Gray items (A): genes upregulated in COS. Blank items (B): genes downregulated in LSW177. Gray items (B): genes downregulated in COS. Figure S3. PCA analysis to examine the similarity between samples. “C” (green) and “L” (blue) represent COS and LSW177, respectively. The numbers after “C” and “L” represent the number of days after pollination. (DOCX 903 kb) [file 12864_2016_3442_MOESM5_ESM.docx]
