# Supplementary material for: Comparative transcriptome analysis of two contrasting watermelon genotypes during fruit development and ripening
Source: BMC Genomics. 2017 Jan 3;18:3. doi: 10.1186/s12864-016-3442-3 (PMC5209866; doi:10.1186/s12864-016-3442-3)
Supplement: Additional file 9: — Summary information and sequence of the LCYB gene in 20 watermelon accessions. (DOCX 19 kb) [file 12864_2016_3442_MOESM9_ESM.docx]

**Additional file 9.** Summary information and sequence of the *LCYB* gene in 20 watermelon accessions [6]

| Accession_Name | Type | Gene Name | Gene Id | Length(bp) | SRA accession |
| --- | --- | --- | --- | --- | --- |
| JX-2 | Cultivar | LCYB | Cla005011 | 1540 | SRR494422 |
| JXF | Cultivar | LCYB | Cla005011 | 1540 | SRR494424 |
| RZ-901 | Cultivar | LCYB | Cla005011 | 1540 | SRR494425 |
| 97103 | Cultivar | LCYB | Cla005011 | 1540 | SRR494426 |
| XHBFGM | Cultivar | LCYB | Cla005011 | 1540 | SRR494427 |
| Black_Diamond | Cultivar | LCYB | Cla005011 | 1540 | SRR494428 |
| Calhoun_Gray | Cultivar | LCYB | Cla005011 | 1540 | SRR494429 |
| Sugarlee | Cultivar | LCYB | Cla005011 | 1540 | SRR494430 |
| Sy-904304 | Cultivar | LCYB | Cla005011 | 1540 | SRR494431 |
| RZ-900 | Cultivar | LCYB | Cla005011 | 1540 | SRR494432 |
| PI482271 | Semi wild | LCYB | Cla005011 | 1540 | SRR494434 |
| PI189317 | Semi wild | LCYB | Cla005011 | 1540 | SRR494441 |
| PI500301 | Semi wild | LCYB | Cla005011 | 1540 | SRR494444 |
| PI595203 | Semi wild | LCYB | Cla005011 | 1540 | SRR494439 |
| PI249010 | Semi wild | LCYB | Cla005011 | 1540 | SRR494443 |
| PI248178 | Semi wild | LCYB | Cla005011 | 1540 | SRR494446 |
| PI482276 | Wild | LCYB | Cla005011 | 1540 | SRR494438 |
| PI482303 | Wild | LCYB | Cla005011 | 1540 | SRR494442 |
| PI296341-FR | Wild | LCYB | Cla005011 | 1540 | SRR494435 |
| PI482326 | Wild | LCYB | Cla005011 | 1540 | SRR494445 |

# JX-2

TTAATCTCTATCCTTTACCAGATTGCCGATCATGTTTACCAAAGATGGAGTTCCCTTTGC

CATGATTTCAAGCCTGGAGGCATTAGATGCGTGAGAGAATAAGGAAAGCCCAAAGAGTAA

CAGCTCAGGAAGGAATAGTCGTGATGACAAGAATCCATGCCAATAACGAGGTTCAAGATC

AAAAAATGCATCAAAAAACCTTCTTGTACCCTTTAGATCCAGCTTCAATAAAATATCCAT

CCCAAAACAGAAAAATTCTCTCTGCCTCCTCCTTTCGATGGGCCATAGATCTTTCCAAAC

TTCAGAGGATATCGCATCACCCCTGAAACGTCCATCTGAACCAAGGCACCGGACTATTGC

ACTAGCAACAATAGGTGCCGCTGCTAGAGTTCTTGCTACCATATATCCAGTTGAAGGGTG

CACCATCCCTGCTGTTCCACCAATTCCAACAACTCTTTGAGGAAGAACTGGCAGCGGTCC

ACCCATTGGAATGACACAATGCTCATCCTCTTCAATGCTCTTCACTTTTATTCCCAAGTG

CTTCAATCTTACCTCCATTCTTTCCTGGATATCGCTCATTTGTAACCCAGGTCGAGCTAC

CAAAGAAGTTTCCTCCAGAAATATCCGATTTGATGAAAAGGGCATTGCATAGAGAAATGT

AGGAATTTTGCTATTTCTCTCCTTCAAAATCATATTGTTATTCAGATGTGAATCTCTCCA

GTCCATAAACACCATCTTGTTAACATCAAATGGATGTTCCTCCACCTCAGCTAAAATCCC

ATAAGCTACCTGGTAGCCTGGATTGTAAGGCTTATCATATTGGACAAGGCATCGAGAGAC

GCCAGTGGCATCAAGAACAATGGCAGCTTGAATGGTCACACCATCATTGCAAATTAACAA

GGATTTGAACTCCTCATGTATAACTTTAATAACTTTAGCTTCATGAAACTTAACACCATT

GGAAATGCATTTCTGCAACATTTTTGACTTGAGTTGCTTTCTATTAACCCTCGCATAAGG

TCGAGCAAGATCTTTTGTTGATTGCTCATTGGTGAACACGACAGCACCAGACCAAGTCGT

GTCGAGACAATCTAGCAAATCCATTGCCTCAAATTCATCCACCCAAACCCCATAATTGTT

GGGCCAAATCAACTTGGGAGATGGGTCAATTGCACAAACTGAAAGCCCTGCCTCTGAAAC

CTGTTGCGCAACAGCAAGCCCTGCTGGGCCGCCTCCCACGACCGCAAGATCGACAACAAG

GCCCTTCGAAGGATCATACATGGGAAGTTCAACCTCAAGATTCTCCTTCTTGGTTTCAGG

AACAAGCTCCAAAAGAGAACTACTTCTCACATTAAGACAACCCCCTTTCCTCCATTTCAG

ACGACCCTTCCTATGACCAAACCCAAATTCCTGACTCTGAAACTTTGTACTCCTCACACC

ACTCACTTTTTCCGAAACCCCATGTAATGGTTGCAGAAAACCATACTTGTTATTGATTTT

AAGTAAAGTATCCAT

# JXF

TTAATCTCTATCCTTTACCAGATTGCCGATCATGTTTACCAAAGATGGAGTTCCCTTTGC

CATGATTTCAAGCCTGGAGGCATTAGATGCGTGAGAGAATAAGGAAAGCCCAAAGAGTAA

CAGCTCAGGAAGGAATAGTCGTGATGACAAGAATCCATGCCAATAACGAGGTTCAAGATC

AAAAAATGCATCAAAAAACCTTCTTGTACCCTTTAGATCCAGCTTCAATAAAATATCCAT

CCCAAAACAGAAAAATTCTCTCTGCCTCCTCCTTTCGATGGGCCATAGATCTTTCCAAAC

TTCAGAGGATATCGCATCACCCCTGAAACGTCCATCTGAACCAAGGCACCGGACTATTGC

ACTAGCAACAATAGGTGCCGCTGCTAGAGTTCTTGCTACCATATATCCAGTTGAAGGGTG

CACCATCCCTGCTGTTCCACCAATTCCAACAACTCTTTGAGGAAGAACTGGCAGCGGTCC

ACCCATTGGAATGACACAATGCTCATCCTCTTCAATGCTCTTCACTTTTATTCCCAAGTG

CTTCAATCTTACCTCCATTCTTTCCTGGATATCGCTCATTTGTAACCCAGGTCGAGCTAC

CAAAGAAGTTTCCTCCAGAAATATCCGATTTGATGAAAAGGGCATTGCATAGAGAAATGT

AGGAATTTTGCTATTTCTCTCCTTCAAAATCATATTGTTATTCAGATGTGAATCTCTCCA

GTCCATAAACACCATCTTGTTAACATCAAATGGATGTTCCTCCACCTCAGCTAAAATCCC

ATAAGCTACCTGGTAGCCTGGATTGTAAGGCTTATCATATTGGACAAGGCATCGAGAGAC

GCCAGTGGCATCAAGAACAATGGCAGCTTGAATGGTCACACCATCATTGCAAATTAACAA

GGATTTGAACTCCTCATGTATAACTTTAATAACTTTAGCTTCATGAAACTTAACACCATT

GGAAATGCATTTCTGCAACATTTTTGACTTGAGTTGCTTTCTATTAACCCTCGCATAAGG

TCGAGCAAGATCTTTTGTTGATTGCTCATTGGTGAACACGACAGCACCAGACCAAGTCGT

GTCGAGACAATCTAGCAAATCCATTGCCTCAAATTCATCCACCCAAACCCCATAATTGTT

GGGCCAAATCAACTTGGGAGATGGGTCAATTGCACAAACTGAAAGCCCTGCCTCTGAAAC

CTGTTGCGCAACAGCAAGCCCTGCTGGGCCGCCTCCCACGACCGCAAGATCGACAACAAG

GCCCTTCGAAGGATCATACATGGGAAGTTCAACCTCAAGATTCTCCTTCTTGGTTTCAGG

AACAAGCTCCAAAAGAGAACTACTTCTCACATTAAGACAACCCCCTTTCCTCCATTTCAG

ACGACCCTTCCTATGACCAAACCCAAATTCCTGACTCTGAAACTTTGTACTCCTCACACC

ACTCACTTTTTCCGAAACCCCATGTAATGGTTGCAGAAAACCATACTTGTTATTGATTTT

AAGTAAAGTATCCAT

# XHBFGM

TTAATCTCTATCCTTTACCAGATTGCCGATCATGTTTACCAAAGATGGAGTTCCCTTTGC

CATGATTTCAAGCCTGGAGGCATTAGATGCGTGAGAGAATAAGGAAAGCCCAAAGAGTAA

CAGCTCAGGAAGGAATAGTCGTGATGACAAGAATCCATGCCAATAACGAGGTTCAAGATC

AAAAAATGCATCAAAAAACCTTCTTGTACCCTTTAGATCCAGCTTCAATAAAATATCCAT

CCCAAAACAGAAAAATTCTCTCTGCCTCCTCCTTTCGATGGGCCATAGATCTTTCCAAAC

TTCAGAGGATATCGCATCACCCCTGAAACGTCCATCTGAACCAAGGCACCGGACTATTGC

ACTAGCAACAATAGGTGCCGCTGCTAGAGTTCTTGCTACCATATATCCAGTTGAAGGGTG

CACCATCCCTGCTGTTCCACCAATTCCAACAACTCTTTGAGGAAGAACTGCCAGCGGTCC

ACCCATTGGAATGACACAATGCTCATCCTCTTCAATGCTCTTCACTTTTATTCCCAAGTG

CTTCAATCTTACCTCCATTCTTTCCTGGATATCGCTCATTTGTAACCCAGGTCGAGCTAC

CAAAGAAGTTTCCTCCAGAAATATCCGATTTGATGAAAAGGGCATTGCATAGAGAAATGT

AGGAATTTTGCTATTTCTCTCCTTCAAAATCATATTGTTATTCAGATGTGAATCTCTCCA

GTCCATAAACACCATCTTGTTAACATCAAATGGATGTTCCTCCACCTCAGCTAAAATCCC

ATAAGCTACCTGGTAGCCTGGATTGTAAGGCTTATCATATTGGACAAGGCATCGAGAGAC

GCCAGTGGCATCAAGAACAATGGCAGCTTGAATGGTCACACCATCATTGCAAATTAACAA

GGATTTGAACTCCTCATGTATAACTTTAATAACTTTAGCTTCATGAAACTTAACACCATT

GGAAATGCATTTCTGCAACATTTTTGACTTGAGTTGCTTTCTATTAACCCTCGCATAAGG

TCGAGCAAGATCTTTTGTTGATTGCTCATTGGTGAACACGACAGCACCAGACCAAGTCGT

GTCGAGACAATCTAGCAAATCCATTGCCTCAAATTCATCCACCCAAACCCCATAATTGTT

GGGCCAAATCAACTTGGGAGATGGGTCAATTGCACAAACTGAAAGCCCTGCCTCTGAAAC

CTGTTGCGCAACAGCAAGCCCTGCTGGGCCGCCTCCCACGACCGCAAGATCGACAACAAG

GCCCTTCGAAGGATCATACATGGGAAGTTCAACCTCAAGATTCTCCTTCTTGGTTTCAGG

AACAAGCTCCAAAAGAGAACTACTTCTCACATTAAGACAACCCCCTTTCCTCCATTTCAG

ACGACCCTTCCTATGACCAAACCCAAATTCCTGACTCTGAAACTTTGTACTCCTCACACC

ACTCACTTTTGCCGAAACCCCATGTAATGGTTGCAGAAAACCATACTTGTTATTGATTTT

AAGTAAAGTATCCAT

# 97103

TTAATCTCTATCCTTTACCAGATTGCCGATCATGTTTACCAAAGATGGAGTTCCCTTTGC

CATGATTTCAAGCCTGGAGGCATTAGATGCGTGAGAGAATAAGGAAAGCCCAAAGAGTAA

CAGCTCAGGAAGGAATAGTCGTGATGACAAGAATCCATGCCAATAACGAGGTTCAAGATC

AAAAAATGCATCAAAAAACCTTCTTGTACCCTTTAGATCCAGCTTCAATAAAATATCCAT

CCCAAAACAGAAAAATTCTCTCTGCCTCCTCCTTTCGATGGGCCATAGATCTTTCCAAAC

TTCAGAGGATATCGCATCACCCCTGAAACGTCCATCTGAACCAAGGCACCGGACTATTGC

ACTAGCAACAATAGGTGCCGCTGCTAGAGTTCTTGCTACCATATATCCAGTTGAAGGGTG

CACCATCCCTGCTGTTCCACCAATTCCAACAACTCTTTGAGGAAGAACTGGCAGCGGTCC

ACCCATTGGAATGACACAATGCTCATCCTCTTCAATGCTCTTCACTTTTATTCCCAAGTG

CTTCAATCTTACCTCCATTCTTTCCTGGATATCGCTCATTTGTAACCCAGGTCGAGCTAC

CAAAGAAGTTTCCTCCAGAAATATCCGATTTGATGAAAAGGGCATTGCATAGAGAAATGT

AGGAATTTTGCTATTTCTCTCCTTCAAAATCATATTGTTATTCAGATGTGAATCTCTCCA

GTCCATAAACACCATCTTGTTAACATCAAATGGATGTTCCTCCACCTCAGCTAAAATCCC

ATAAGCTACCTGGTAGCCTGGATTGTAAGGCTTATCATATTGGACAAGGCATCGAGAGAC

GCCAGTGGCATCAAGAACAATGGCAGCTTGAATGGTCACACCATCATTGCAAATTAACAA

GGATTTGAACTCCTCATGTATAACTTTAATAACTTTAGCTTCATGAAACTTAACACCATT

GGAAATGCATTTCTGCAACATTTTTGACTTGAGTTGCTTTCTATTAACCCTCGCATAAGG

TCGAGCAAGATCTTTTGTTGATTGCTCATTGGTGAACACGACAGCACCAGACCAAGTCGT

GTCGAGACAATCTAGCAAATCCATTGCCTCAAATTCATCCACCCAAACCCCATAATTGTT

GGGCCAAATCAACTTGGGAGATGGGTCAATTGCACAAACTGAAAGCCCTGCCTCTGAAAC

CTGTTGCGCAACAGCAAGCCCTGCTGGGCCGCCTCCCACGACCGCAAGATCGACAACAAG

GCCCTTCGAAGGATCATACATGGGAAGTTCAACCTCAAGATTCTCCTTCTTGGTTTCAGG

AACAAGCTCCAAAAGAGAACTACTTCTCACATTAAGACAACCCCCTTTCCTCCATTTCAG

ACGACCCTTCCTATGACCAAACCCAAATTCCTGACTCTGAAACTTTGTACTCCTCACACC

ACTCACTTTTTCCGAAACCCCATGTAATGGTTGCAGAAAACCATACTTGTTATTGATTTT

AAGTAAAGTATCCAT

# Black_Diamond

TTAATCTCTATCCTTTACCAGATTGCCGATCATGTTTACCAAAGATGGAGTTCCCTTTGC

CATGATTTCAAGCCTGGAGGCATTAGATGCGTGAGAGAATAAGGAAAGCCCAAAGAGTAA

CAGCTCAGGAAGGAATAGTCGTGATGACAAGAATCCATGCCAATAACGAGGTTCAAGATC

AAAAAATGCATCAAAAAACCTTCTTGTACCCTTTAGATCCAGCTTCAATAAAATATCCAT

CCCAAAACAGAAAAATTCTCTCTGCCTCCTCCTTTCGATGGGCCATAGATCTTTCCAAAC

TTCAGAGGATATCGCATCACCCCTGAAACGTCCATCTGAACCAAGGCACCGGACTATTGC

ACTAGCAACAATAGGTGCCGCTGCTAGAGTTCTTGCTACCATATATCCAGTTGAAGGGTG

CACCATCCCTGCTGTTCCACCAATTCCAACAACTCTTTGAGGAAGAACTGGCAGCGGTCC

ACCCATTGGAATGACACAATGCTCATCCTCTTCAATGCTCTTCACTTTTATTCCCAAGTG

CTTCAATCTTACCTCCATTCTTTCCTGGATATCGCTCATTTGTAACCCAGGTCGAGCTAC

CAAAGAAGTTTCCTCCAGAAATATCCGATTTGATGAAAAGGGCATTGCATAGAGAAATGT

AGGAATTTTGCTATTTCTCTCCTTCAAAATCATATTGTTATTCAGATGTGAATCTCTCCA

GTCCATAAACACCATCTTGTTAACATCAAATGGATGTTCCTCCACCTCAGCTAAAATCCC

ATAAGCTACCTGGTAGCCTGGATTGTAAGGCTTATCATATTGGACAAGGCATCGAGAGAC

GCCAGTGGCATCAAGAACAATGGCAGCTTGAATGGTCACACCATCATTGCAAATTAACAA

GGATTTGAACTCCTCATGTATAACTTTAATAACTTTAGCTTCATGAAACTTAACACCATT

GGAAATGCATTTCTGCAACATTTTTGACTTGAGTTGCTTTCTATTAACCCTCGCATAAGG

TCGAGCAAGATCTTTTGTTGATTGCTCATTGGTGAACACGACAGCACCAGACCAAGTCGT

GTCGAGACAATCTAGCAAATCCATTGCCTCAAATTCATCCACCCAAACCCCATAATTGTT

GGGCCAAATCAACTTGGGAGATGGGTCAATT---------------CCTGCCGCTGAAAC

CTGTTGCGCAACAGCAAGCCCTGCTGGGCCGCCTCCCACGACCGCAAGATCGACAACAAG

GCCCTTCGAAGGATCATACATGGGAAGTTCAACCTCAAGATTCTCCTTCTTGGTTTCAGG

AACAAGCTCCAAAAGAGAACTACTTCTCACATTAAGACAACCCCCTTTCCTCCATTTCAG

ACGACCCTTCCTATGACCAAACCCAAATTCCTGACTCTGAAACTTTGTACTCCTCACACC

ACTCACTTTTTCCGAAACCCCATGTAATGGTTGCAGAAAACCATACTTGTTATTGATTTT

AAGTAAAGTATCCAT

# Calhoun_Gray

TTAATCTCTATCCTTTACCAGATTGCCGATCATGTTTACCAAAGATGGAGTTCCCTTTGC

CATGATTTCAAGCCTGGAGGCATTAGATGCGTGAGAGAATAAGGAAAGCCCAAAGAGTAA

CAGCTCAGGAAGGAATAGTCGTGATGACAAGAATCCATGCCAATAACGAGGTTCAAGATC

AAAAAATGCATCAAAAAACCTTCTTGTACCCTTTAGATCCAGCTTCAATAAAATATCCAT

CCCAAAACAGAAAAATTCTCTCTGCCTCCTCCTTTCGATGGGCCATAGATCTTTCCAAAC

TTCAGAGGATATCGCATCACCCCTGAAACGTCCATCTGAACCAAGGCACCGGACTATTGC

ACTAGCAACAATAGGTGCCGCTGCTAGAGTTCTTGCTACCATATATCCAGTTGAAGGGTG

CACCATCCCTGCTGTTCCACCAATTCCAACAACTCTTTGAGGAAGAACTGGCAGCGGTCC

ACCCATTGGAATGACACAATGCTCATCCTCTTCAATGCTCTTCACTTTTATTCCCAAGTG

CTTCAATCTTACCTCCATTCTTTCCTGGATATCGCTCATTTGTAACCCAGGTCGAGCTAC

CAAAGAAGTTTCCTCCAGAAATATCCGATTTGATGAAAAGGGCATTGCATAGAGAAATGT

AGGAATTTTGCTATTTCTCTCCTTCAAAATCATATTGTTATTCAGATGTGAATCTCTCCA

GTCCATAAACACCATCTTGTTAACATCAAATGGATGTTCCTCCACCTCAGCTAAAATCCC

ATAAGCTACCTGGTAGCCTGGATTGTAAGGCTTATCATATTGGACAAGGCATCGAGAGAC

GCCAGTGGCATCAAGAACAATGGCAGCTTGAATGGTCACACCATCATTGCAAATTAACAA

GGATTTGAACTCCTCATGTATAACTTTAATAACTTTAGCTTCATGAAACTTAACACCATT

GGAAATGCATTTCTGCAACATTTTTGACTTGAGTTGCTTTCTATTAACCCTCGCATAAGG

TCGAGCAAGATCTTTTGTTGATTGCTCATTGGTGAACACGACAGCACCAGACCAAGTCGT

GTCGAGACAATCTAGCAAATCCATTGCCTCAAATTCATCCACCCAAACCCCATAATTGTT

GGGCCAAATCAACTTGGGAGATGGGTCAATTGCACAAACTGAAACCCCTGCCTCTGAAAC

CTGTTGCGCAACAGCAAGCCCTGCTGGGCCGCCTCCCACGACCGCAAGATCGACAACAAG

GCCCTTCGAAGGATCATACATGGGAAGTTCAACCTCAAGATTCTCCTTCTTGGTTTCAGG

AACAAGCTCCAAAAGAGAACTACTTCTCACATTAAGACAACCCCCTTTCCTCCATTTCAG

ACGACCCTTCCTATGACCAAACCCAAATTCCTGACTCTGAAACTTTGTACTCCTCACACC

ACTCACTTTTTCCGAAACCCCATGTAATGGTTGCAGAAAACCATACTTGTTATTGATTTT

AAGTAAAGTATCCAT

# PI595203

TTAATCTCTATCCTTTACCAGATTGCCGATCATGTTTACCAAAGATGGAGTTCCCTTTGC

CATGATTTCAAGCCTGGAGGCATTAGATGCGTGAGAGAATAAGGAAAGCCCAAAGAGTAA

CAGCTCAGGAAGGAATAGTCGTGATGACAAGAATCCATGCCAATAACGAGGTTCAAGATC

AAAAAATGCATCAAAAAACCTTCTTGTACCCTTTAGATCCAGCTTCAATAAAATATCCAT

CCCAAAACAGAAAAATTCTCTCTGCCTCCTCCTTTCGATGGGCCATAGATCTTTCCAAAC

TTCAGAGGATATCGCATCACCCCTGAAACGTCCATCTGAACCAAGGCACCGGACTATTGC

ACTAGCAACAATAGGTGCCGCTGCTA----------------------AGTTGAAGGGTG

CACCATCCCTGCTGTTCCACCAATTCCAACAACTCTTTGAGGAAGAACTGGCAGCGGTCC

ACCCATTGGAATGACACAATGCTCATCCTCTTCAATGCTCTTCACTTTTATTCCCAAGTG

CTTCAATCTTACCTCCATTCTTTCCTGGATATCGCTCATTTGTAACCCAGGTCGAGCTAC

CAAAGAAGTTTCCTCCAGAAATATCCGATTTGATGAAAAGGGCATTGCATAGAGAAATGT

AGGAATTTTGCTATTTCTCTCCTTCAAAATCATATTGTTATTCAGATGTGAATCTCTCCA

GTCCATAAACACCATCTTGTTAACATCAAATGGATGTTCCTCCCCCTCAGCTAAAATCCC

ATAAGCTACCTGGTAGCCTGGATTGTAAGGCTTATCATATTGGACAAGGCATCGAGAGAA

GCCAGTGGCATCAAGAACAATGGCAGCTTGAATGGTCACACCATCATCGCAA-------A

GGATTTGAACTCCTCATGTATAACTTTAATAACTTTAGCTTCATGAAACTTAACACCATT

GGAAATGCATTTCTGCAACATTTTTGACTTGAGTTGCTTTCTATTAACCCTCGCATAAGG

TCGAGCAAGATCTTTTGTTGATTGCTCATTGGTGAACACGACAGCACCAGACCAAGTCGT

GTCGAGACAATCTAGCAAATCCA---------ATTCATCCACCCAAACCCCATAATTGTT

GGGCCAAATCAACTTG-------------TTGCACAAACTGAAAGCCCTGCCTCTGAAAC

CTGTTGCGCAACAGCAAGCCCGGCTGGGCCGCCTCCCACGACCGCAAGATCGACAACAAG

GCCCTTCGAAGGATCATACATGGGAAGTTCAACCTCAAGATTCTCCTTCTTGGTTTCAGG

AACAAGCTCCAAAAGAGAACTACTTCTCACATTAAGACAACCCCCTTTCCTCCATTTCAG

ACGACCCTTCCTATGACCAAACCCAAATTCCTGACTCTGAAACTTTGTACTCCTCACACC

ACTCACTTTTTCCGAAACCCCATGTAATGGTTGCAGAAAACCATACTTGTTATTGATTTT

AAGCAAAGTATCCAT

# PI249010

TTAATCTCTATCCTTTACCAGATTGCCGATCATGTTTACCAAAGATGGAGTTCCCTTTGC

CATGATTTCAAGCCTGGAGGCATTAGATGCGTGAGAGAATAAGGAAAGCCCAAAGAGTAA

CAGCTCAGGAAGGAATAGTCGTGATGACAAGAATCCATGCCAATAACGAGGTTCAAGATC

AAAAAATGCATCAAAAAACCTTCTTGTACCCTTTAGATCCAGCTTCAATAAAATATCCAT

CCCAAAACAGAAAAATTCTCTCTGCCTCCTCCTTTCGATGGGCCATAGATCTTTCCAAAC

TTCAGAGGATATCGCATCACCCCTGAAACGTCCATCTGAACCAAGGCACCGGACTATTGC

ACTAGCAACAATAGGTGCCGCTGCTAGAGTTCTTGCTACCATATATCCAGTTGAAGGGTG

CACCATCCCTGCTGTTCCACCAATTCCAACAACTCTTTGAGGAAGAACTGGCAGCGGTCC

ACCCATTGGAATGACACAATGCTCATCCTCTTCAATGCTCTTCACTTTTATTCCCAAGTG

CTTCAATCTTACCTCCATTCTTTCCTGGATATCGCTCATTTGTAACCCAGGTCGAGCTAC

CAAAGAAGTTTCCTCCAGAAATATCCGATTTGATGAAAAGGGCATTGCATAGAGAAATGT

AGGAATTTTGCTATTTCTCTCCTTCAAAATCATATTGTTATTCAGATGTGAATCTCTCCA

GTCCATAAACACCATCTTGTTAACATCAAATGGATGTTCCTCCACCTCAGCTAAAATCCC

ATAAGCTACCTGGTAGCCTGGATTGTAAGGCTTATCATATTGGACAAGGCATCGAGAGAA

GCCAGTGGCATCAAGAACAATGGCAGCTTGAATGGTCACACCATCATTGCAAATTAACAA

GGATTTGAACTCCTCATGTATAACTTTAATAACTTTAGCTTCATGAAACTTAACACCATT

GGAAATGCATTTCTGCAACATTTTTGACTTGAGTTGCTTTCTATTAACCCTCGCATAAGG

TCGAGCAAGATCTTTTGTTGATTGCTCATTGGTGAACACGACAGCACCAGACCAAGTCGT

GTCGAGACAATCTAGCAAATCCATTGCCTCAAATTCATCCACCCAAACCCCATAATTGTT

GGGCCAAATCAACTTGGGAGATGGGTCAATTGCACAAACTGAAAGCCCTGCCTCTGAAAC

CTGTTGCGCAACAGCAAGCCCTGCTGGGCCGCCTCCCACGACCGCAAGATCGACAACAAG

GCCCTTCGAAGGATCATACATGGGAAGTTCAACCTCAAGATTCTCCTTCTTGGTTTCAGG

AACAAGCTCCAAAAGAGAACTACTTCTCACATTAAGACAACCCCCTTTCCTCCATTTCAG

ACGACCCTTCCTATGACCAAACCCAAATTCCTGACTCTGAAACTTTGTACTCCTCACACC

ACTCACTTTTTCCGAAACCCCATGTAATGGTTGCAGAAAACCATACTTGTTATTGATTTT

AAGTAAAGTATCCAT

# PI189317

TTAATCTCTATCCTTTACCAGATTGCCGATCATGTTTACCAAAGATGGAGTTCCCTTTGC

CATGATTTCAAGCCTGGAGGCATTAGATGCGTGAGAGAATAAGGAAAGCCCAAAGAGTAA

CAGCTCAGGAAGGAATAGTCGTGATGACAAGAATCCATGCCAATAACGAGGTTCAAGATC

AAAAAATGCATCAAAAAACCTTCTTGTACCCTTTAGATCCAGCTTCAATAAAATATCCAT

CCCAAAACAGAAAAATTCTCTCTGCCTCCTCCTTTCGATGGGCCATAGATCTTTCCAAAC

TTCAGAGGATATCGCATCACCCCTGAAACGTCCATCTGAACCAAGGCACCGGACTATTGC

ACTAGCAACAATAGGTGCCGCTGCTAGAGTTCTTGCTACCATATATCCAGTTGAAGGGTG

CACCATCCCTGCTGTTCCACCAATTCCAACAACTCTTTGAGGAAGAACTGGCAGCGGTCC

ACCCATTGGAATGACACAATGCTCATCCTCTTCAATGCTCTTCACTTTTATTCCCAAGTG

CTTCAATCTTACCTCCATTCTTTCCTGGATATCGCTCATTTGTAACCCAGGTCGAGCTAC

CAAAGAAGTTTCCTCCAGAAATATCCGATTTGATGAAAAGGGCATTGCATAGAGAAATGT

AGGAATTTTGCTATTTCTCTCCTTCAAAATCATATTGTTATTCAGATGTGAATCTCTCCA

GTCCATAAACACCATCTTGTTAACATCAAATGGATGTTCCTCCACCTCAGCTAAAATCCC

ATAAGCTACCTGGTAGCCTGGATTGTAAGGCTTATCATATTGGACAAGGCATCGAGAGAC

GCCAGTGGCATCAAGAACAATGGCAGCTTGAATGGTCACACCATCATTGCAAATTAACAA

GGATTTGAACTCCTCATGTATAACTTTAATAACTTTAGCTTCATGAAACTTAACACCATT

GGAAATGCATTTCTGCAACATTTTTGACTTGAGTTGCTTTCTATTAACCCTCGCATAAGG

TCGAGCAAGATCTTTTGTTGATTGCTCATTGGTGAACACGACAGCACCAGACCAAGTCGT

GTCGAGACAATCTAGCAAATCCATTGCCTCAAATTCATCCACCCAAACCCCATAATTGTT

GGGCCAAATCAACTTGGGAGATGGGTCAATTGCACAAACTGAAAGCCCTGCCTCTGAAAC

CTGTTGCGCAACAGCAAGCCCTGCTGGGCCGCCTCCCACGACCGCAAGATCGACAACAAG

GCCCTTCGAAGGATCATACATGGGAAGTTCAACCTCAAGATTCTCCTTCTTGGTTTCAGG

AACAAGCTCCAAAAGAGAACTACTTCTCACATTAAGACAACCCCCTTTCCTCCATTTCAG

ACGACCCTTCCTATGACCAAACCCAAATTCCTGACTCTGAAACTTTGTACTCCTCACACC

ACTCACTTTTTCCGAAACCCCATGTAATGGTTGCAGAAAACCATACTTGTTATTGATTTT

AAGTAAAGTATCCAT

# PI500301

TTAATCTCTATCCTTTACCAGATTGCCGATCATGTTTACCAAAGATGGAGTTCCCTTTGC

CATGATTTCAAGCCTGGAGGCATTAGATGCGTGAGAGAATAAGGAAAGCCCAAAGAGTAA

CAGCTCAGGAAGGAATAGTCGTGATGACAAGAATCCATGCCAATAACGAGGTTCAAGATC

AAAAAATGCATCAAAAAACCTTCTTGTACCCTTTAGATCCAGCTTCAATAAAATATCCAT

CCCAAAACAGAAAAATTCTCTCTGCCTCCTCCTTTCGATGGGCCATAGATCTTTCCAAAC

TTCAGAGGATATCGCATCACCCCTGAAACGTCCATCTGAACCAAGGCACCGGACTATTGC

ACTAGCAACAATAGGTGCCGCTGCTAGAGTTCTTGCTACCATATATCCAGTTGAAGGGTG

CACCATCCCTGCTGTTCCACCAATTCCAACAACTCTTTGAGGAAGAACTGGCAGCGGTCC

ACCCATTGGAATGACACAATGCTCATCCTCTTCAATGCTCTTCACTTTTATTCCCAAGTG

CTTCAATCTTACCTCCATTCTTTCCTGGATATCGCTCATTTGTAACCCAGGTCGAGCTAC

CAAAGAAGTTTCCTCCAGAAATATCCGATTTGATGAAAAGGGCATTGCATAGAGAAATGT

AGGAATTTTGCTATTTCTCTCCTTCAAAATCATATTGTTATTCAGATGTGAATCTCTCCA

GTCCATAAACACCATCTTGTTAACATCAAATGGATGTTCCTCCACCTCAGCTAAAATCCC

ATAAGCTACCTGGTAGCCTGGATTGTAAGGCTTATCATATTGGACAAGGCATCGAGAGAC

GCCAGTGGCATCAAGAACAATGGCAGCTTGAATGGTCACACCATCATTGCAAATTAACAA

GGATTTGAACTCCTCATGTATAACTTTAATAACTTTAGCTTCATGAAACTTAACACCATT

GGAAATGCATTTCTGCAACATTTTTGACTTGAGTTGCTTTCTATTAACCCTCGCATAAGG

TCGAGCAAGATCTTTTGTTGATTGCTCATTGGTGAACACGACAGCACCAGACCAAGTCGT

GTCGAGACAATCTAGCAAATCCATTGCCTCAAATTCATCCACCCAAACCCCATAATTGTT

GGGCCAAATCAACTTGGGAGATGGGTCAATTGCACAAACTGAAAGCCCTGCCTCTGAAAC

CTGTTGCGCAACAGCAAGCCCTGCTGGGCCGCCTCCCACGACCGCAAGATCGACAACAAG

GCCCTTCGAAGGATCATACATGGGAAGTTCAACCTCAAGATTCTCCTTCTTGGTTTCAGG

AACAAGCTCCAAAAGAGAACTACTTCTCACATTAAGACAACCCCCTTTCCTCCATTTCAG

ACGACCCTTCCTATGACCAAACCCAAATTCCTGACTCTGAAACTTTGTACTCCTCACACC

ACTCACTTTTTCCGAAACCCCATGTAATGGTTGCAGAAAACCATACTTGTTATTGATTTT

AAGTAAAGTATCCAT

# PI248178

TTAATCTCTATCCTTTACCAGATTGCCGATCATGTTTACCAAAGATGGAGTTCCCTTTGC

CATGATTTCAAGCCTGGAGGCATTAGATGCGTGAGAGAATAAGGAAAGCCCAAAGAGTAA

CAGCTCAGGAAGGAATAGTCGTGATGACAAGAATCCATGCCAATAACGAGGTTCAAGATC

AAAAAATGCATCAAAAAACCTTCTTGTACCCTTTAGATCCAGCTTCAATAAAATATCCAT

CCCAAAACAGAAAAATTCTCTCTGCCTCCTCCTTTCGATGGGCCATAGATCTTTCCAAAC

TTCAGAGGATATCGCATCACCCCTGAAACGTCCATCTGAACCAAGGCACCGGACTATTGC

ACTAGCAACAATAGGTGCCGCTGCTAGAGTTCTTGCTACCATATATCCAGTTGAAGGGTG

CACCATCCCTGCTGTTCCACCAATTCCAACAACTCTTTGAGGAAGAACTGGCAGCGGTCC

ACCCATTGGAATGACACAATGCTCATCCTCTTCAATGCTCTTCACTTTTATTCCCAAGTG

CTTCAATCTTACCTCCATTCTTTCCTGGATATCGCTCATTTGTAACCCAGGTCGAGCTAC

CAAAGAAGTTTCCTCCAGAAATATCCGATTTGATGAAAAGGGCATTGCATAGAGAAATGT

AGGAATTTTGCTATTTCTCTCCTTCAAAATCATATTGTTATTCAGATGTGAATCTCTCCA

GTCCATAAACACCATCTTGTTAACATCAAATGGATGTTCCTCCACCTCAGCTAAAATCCC

ATAAGCTACCTGGTAGCCTGGATTGTAAGGCTTATCATATTGGACAAGGCATCGAGAGAC

GCCAGTGGCATCAAGAACAATGGCAGCTTGAATGGTCACACCATCATTGCAAATTAACAA

GGATTTGAACTCCTCATGTATAACTTTAATAACTTTAGCTTCATGAAACTTAACACCATT

GGAAATGCATTTCTGCAACATTTTTGACTTGAGTTGCTTTCTATTAACCCTCGCATAAGG

TCGAGCAAGATCTTTTGTTGATTGCTCATTGGTGAACACGACAGCACCAGACCAAGTCGT

GTCGAGACAATCTAGCAAATCCATTGCCTCAAATTCATCCACCCAAACCCCATAATTGTT

GGGCCAAATCAACTTGGGAGATGGGTCAATTGCACAAACTGAAAGCCCTGCCTCTGAAAC

CTGTTGCGCAACAGCAAGCCCTGCTGGGCCGCCTCCCACGACCGCAAGATCGACAACAAG

GCCCTTCGAAGGATCATACATGGGAAGTTCAACCTCAAGATTCTCCTTCTTGGTTTCAGG

AACAAGCTCCAAAAGAGAACTACTTCTCACATTAAGACAACCCCCTTTCCTCCATTTCAG

ACGACCCTTCCTATGACCAAACCCAAATTCCTGACTCTGAAACTTTGTACTCCTCACACC

ACTCACTTTTTCCGAAACCCCATGTAATGGTTGCAGAAAACCATACTTGTTATTGATTTT

AAGTAAAGTATCCAT

# PI482271

TTAATCTCTATCCTTTACCAGATTGCCGATCATGTTTACCAAAGATGGAGTTCCCTTTGC

CATGATTTCAAGCCTGGAGGCATTAGATGCGTGAGAGAATAAGGAAAGCCCAAAGAGTAA

CAGCTCAGGAAGGAATAGTCGTGATGACAAGAATCCATGCCAATAACGAGGTTCAAGATC

AAAAAATGCATCAAAAAACCTTCTTGTACCCTTTAGATCCAGCTTCAATAAAATATCCAT

CCCAAAACAGAAAAATTCTCTCTGCCTCCTCCTTTCGATGGGCCATAGATCTTTCCAAAC

TTCAGAGGATATCGCATCACCCCTGAAACGTCCATCTGAACCAAGGCACCGGACTATTGC

ACTAGCAACAATAGGTGCCGCTGCTAGAGTTCTTGCTACCATATATCCAGTTGAAGGGTG

CACCATCCCTGCTGTTCCACCAATTCCAACAACTCTTTGAGGAAGAACTGGCAGCGGTCC

ACCCATTGGAATGACACAATGCTCATCCTCTTCAATGCTCTTCACTTTTATTCCCAAGTG

CTTCAATCTTACCTCCATTCTTTCCTGGATATCGCTCATTTGTAACCCAGGTCGAGCTAC

CAAAGAAGTTTCCTCCAGAAATATCCGATTTGATGAAAAGGGCATTGCATAGAGAAATGT

AGGAATTTTGCTATTTCTCTCCTTCAAAATCATATTGTTATTCAGATGTGAATCTCTCCA

GTCCATAAACACCATCTTGTTAACATCAAATGGATGTTCCTCCACCTCAGCTAAAATCCC

ATAAGCTACCTGGTAGCCTGGATTGTAAGGCTTATCATATTGGACAAGGCATCGAGAGAC

GCCAGTGGCATCAAGAACAATGGCAGCTTGAATGGTCACACCATCATTGCAAATTAACAA

GGATTTGAACTCCTCATGTATAACTTTAATAACTTTAGCTTCATGAAACTTAACACCATT

GGAAATGCATTTCTGCAACATTTTTGACTTGAGTTGCTTTCTATTAACCCTCGCATAAGG

TCGAGCAAGATCTTTTGTTGATTGCTCATTGGTGAACACGACAGCACCAGACCAAGTAGT

GTCGAGACAATCAAGCAAATCCATTGCCTCAAATTCATCCACCCAAACCCCATAATTGTT

GGGCCAAATCAACTTGGGAGATGGGTCAATTGCACAAACTGAAAGCCCTGCCTCTGAAAC

CTGTTGCGCAACAGCAAGCCCTGCTGGGCCGCCTCCCACGACCGCAAGATCGACAACAAG

GCCCTTCGAAGGATCATACATGGGAAGTTCAACCTCAAGATTCTCCTTCTTGGTTTCAGG

AACAAGCTCCAAAAGAGAACTACTTCTCACATTAAGACAACCCCCTTTCCTCCATTTCAG

ACGACCCTTCCTATGACCAAACCCAAATTCCTGACTCTGAAACTTTGTACTCCTCACACC

ACTCACTTTTTCCGAAACCCCATGTAATGGTTGCAGAAAACCATACTTGTTATTGATTTT

AAGTAAAGTATCCAT

# PI482276

TTAATCTCTATCCTTTACCAGATTGCCGATCATGTTTACCAAAGATGGAGTTCCCTTTGC

CATGATTTCAAGCCTGGAGGCATTAGATGCGTGAGAGAATAAGGAAAGCCCAAAGAGTAA

CAGCTCAGGAAGGAATAGTCGTGATGA---GAATCCATGCCAATAACGAGGTTCAAGATC

AAAAAATGCATCAAAAAACCTTCTTGTACCCTTTAGATCCAGCTTCAATAAAATATCCAT

CCCAAAACAGAAAAATTCTCTCTGCCTCCTCCTTTCGATGGGCCATAGATCTTTCCAAAC

TTCAGAGGATATCGCATCACCCCTGAAACGTCCATCTGAACCAAGGCACCGGACTATTGC

ACTAGCAACAATAGGTGCTGCTGCTAGAGTTCTTGCTACCATATATCCAGTTGAAGGGTG

CACCATCCCTGCTGTTCCACCAATTCCAACAACTCTTTGAGGAAGAACTGGCAGCGGTCC

ACCCATTGGAATGACACAATGCTCATCCTCTTCAATGCTCTTCACTTTTATTCCCAAGTG

CTTCAATCTTACCTCCATTCTTTCCTGGATATCGCTCATTTGTAAACCAGGTCGAGCTAC

CAAAGAAGTTTCCTCCAGAAATATCCGATTTGATGAAAAGGGCATTGCATAGAGAAATGT

AGGAATTTTGCTATTTCTCTCCTTCAAAATCATATTGTTATTCAGATGCGAATCTCTCCA

GTCCATAAACACCATCTTGTTAACATCAAATGGATGTTCCTCCACCTCAGCTAAAATCCC

ATAAGCTACCTGGTAGCCTGGATTGTAAGGCTTATCATATTGGACAAGGCATCGAGAGAA

GCCAGTAGCATCAAGAACAATGGCAGCTTGAATGGTCACACCATCATTGCAAATTAACAA

GGATTTGAACTCCTCATGTATAACTTTAATAACTTTAGCTTCATGAAACTTAACACCATT

GGAAATGCATTTCTGCAACATTTTTGACTTGAGTTGCTTTCTATTAACCCTCGCATAAGG

TCGAGCAAGATCTTTTGTTGATTGCTCATTGGTGAACACGACAGCGCCAGACCAAGTCGT

GTCGAGACAATCTAGCAAATCCATTGCCTCAAATTCATCCACCCAAACCCCATAATTGTT

GGGCCAAATCAACTTGGGAGATGGGTCAATTGCACAAACTGAAAGCCCTGCCTCTGAAAC

CTGTTGCGCAACAGCAAGCCCTGCTGGGCCGCCTCCCACGACTGCAAGATCGACAACAAG

GCCCTTCGAAGGATCATACATGGGAAGTTCAACCTCAAGATTCTCCTTCTTGGTTTCAGG

AACAAGCTCCAAAAGAGCACTACTTCTCACATTAAGACAACCCCCTTTCCTCCATTTCAG

ACGACCCTTCCTATGACCAAATCCAAATTCCTGACTCTGAAACTTTGTACTCCTCACACC

ACTCACTTTTTCCGAAACCCCATGTAATGGTTGCAGAAAACCATACTTGTTATTGATTTT

AAGCAAAGTATCCAT

# PI482303

TTAATCTCTATCCTTTACCAGATTGCCGATCATGTTTACCAAAGATGGAGTTCCCTTTGC

CATGATTTCAAGCCTGGAGGCATTAGATGCGTGAGAGAATAAGGAAAGCCCAAAGAGTAA

CAGCTCAGGAAGGAATAGTCGTGATGACAAGAATCCATGCCAATAACGAGGTTCAAGATC

AAAAAATGCATCAAAAAACCTTCTTGTACCCTTTAGATCCAGCTTCAATAAAATATCCAT

CCCAAAACAGAAAAATTCTCTCTGCCTCCTCCTTTCGATGGGCCATAGATCTTTCCAAAC

TTCAGAGGATATCGCATCACCCCTGAAACGTCCATCTGAACCAAGGCACCGGACTATTGC

ACTAGCAACAATAGGTGCTGCTGCTAGAGTTCTTGCTACCATATATCCAGTTGAAGGGTG

CACCATCCCTGCTGTTCCACCAATTCCAACAACTCTTTGAGGAAGAACTGGCAGCGGTCC

ACCCATTGGAATGACACAATGCTCATCCTCTTCAATGCTCTTCACTTTTATTCCCAAGTG

CTTCAATCTTACCTCCATTCTTTCCTGGATATCGCTCATTTGTAAACCAGGTCGAGCTAC

CAAAGAAGTTTCCTCCAGAAATATCCGATTTGATGAAAAGGGCATTGCATAGAGAAATGT

AGGAATTTTGCTATTTCTCTCCTTCAAAATCATATTGTTATTCAGATGCGAATCTCTCCA

GTCCATAAACACCATCTTGTTAACATCAAATGGATGTTCCTCCACCTCAGCTAAAATCCC

ATAAGCTACCTGGTAGCCTGGATTGTAAGGCTTATCATATTGGACAAGGCATCGAGAGAA

GCCAGTAGCATCAAGAACAATGGCAGCTTGAATGGTCACACCATCATTGCAAATTAACAA

GGATTTGAACTCCTCATGTATAACTTTAATAACTTTAGCTTCATGAAACTTAACACCATT

GGAAATGCATTTCTGCAACATTTTTGACTTGAGTTGCTTTCTATTAACCCTCGCATAAGG

TCGAGCAAGATCTTTTGTTGATTGCTCATTGGTGAACACGACAGCGCCAGACCAAGTCGT

GTCGAGACAATCTAGCAAATCCATTGCCTCAAATTCATCCACCCAAACCCCATAATTGTT

GGGCCAAATCAACTTGGGAGATGGGTCAATTGCACAAACTGAAAGCCCTGCCTCTGAAAC

CTGTTGCGCAACAGCAAGCCCTGCTGGGCCGCCTCCCACGACTGCAAGATCGACAACAAG

GCCCTTCGAAGGATCATACATGGGAAGTTCAACCTCAAGATTCTCCTTCTTGGTTTCAGG

AACAAGCTCCAAAAGAGCACTACTTCTCACATTAAGACAACCCCCTTTCCTCCATTTCAG

ACGACCCTTCCTATGACCAAATCCAAATTCCTGACTCTGAAACTTTGTACTCCTCACACC

ACTCACTTTTTCCGAAACCCCATGTAATGGTTGCAGAAAACCATACTTGTTATTGATTTT

AAGCAAAGTATCCAT

# PI482326

TTAATCTCTATCCTTTACCAGATTGCCGATCATGTTTACCAAAGATGGAGTTCCCTTTGC

CATGATTTCAAGCCTGGAGGCATTAGATGCGTGAGAGAATAAGGAAAGCCCAAAGAGTAA

CAGCTCAGGAAGGAATAGTCGTGATGACAAGAATCCATGCCAATAACGAGGTTCAAGATC

AAAAAATGCATCAAAAAACCTTCTTGTACCCTTTAGATCCAGCTTCAATAAAATATCCAT

CCCAAAACAGAAAAATTCTCTCTGCCTCCTCCTTTCGATGGGCCATAGATCTTTCCAAAC

TTCAGAGGATATCGCATCACCCCTGAAACGTCCATCTGAACCAAGGCACCGGACTATTGC

ACTAGCAACAATAGGTGCTGCTGCTAGAGTTCTTGCTACCATATATCCAGTTGAAGGGTG

CACCATCCCTGCTGTTCCACCAATTCCAACAACTCTTTGAGGAAGAACTGGCAGCGGTCC

ACCCATTGGAATGACACAATGCTCATCCTCTTCAATGCTCTTCACTTTTATTCCCAAGTG

CTTCAATCTTACCTCCATTCTTTCCTGGATATCGCTCATTTGTAAACCAGGTCGAGCTAC

CAAAGAAGTTTCCTCCAGAAATATCCGATTTGATGAAAAGGGCATTGCATAGAGAAATGT

AGGAATTTTGCTATTTCTCTCCTTCAAAATCATATTGTTATTCAGATGCGAATCTCTCCA

GTCCATAAACACCATCTTGTTAACATCAAATGGATGTTCCTCCACCTCAGCTAAAATCCC

ATAAGCTACCTGGTAGCCTGGATTGTAAGGCTTATCATATTGGACAAGGCATCGAGAGAA

GCCAGTAGCATCAAGAACAATGGCAGCTTGAATGGTCACACCATCATTGCAAATTAACAA

GGATTTGAACTCCTCATGTATAACTTTAATAACTTTAGCTTCATGAAACTTAACACCATT

GGAAATGCATTTCTGCAACATTTTTGACTTGAGTTGCTTTCTATTAACCCTCGCATAAGG

TCGAGCAAGATCTTTTGTTGATTGCTCATTGGTGAACACGACAGCGCCAGACCAAGTCGT

GTCGAGACAATCTAGCAAATCCATTGCCTCAAATTCATCCACCCAAACCCCATAATTGTT

GGGCCAAATCAACTTGGGAGATGGGTCAATTGCACAAACTGAAAGCCCTGCCTCTGAAAC

CTGTTGCGCAACAGCAAGCCCTGCTGGGCCGCCTCCCACGACTGCAAGATCGACAACAAG

GCCCTTCGAAGGATCATACATGGGAAGTTCAACCTCAAGATTCTCCTTCTTGGTTTCAGG

AACAAGCTCCAAAAGAGCACTACTTCTCACATTAAGACAACCCCCTTTCCTCCATTTCAG

ACGACCCTTCCTATGACCAAATCCAAATTCCTGACTCTGAAACTTTGTACTCCTCACACC

ACTCACTTTTTCCGAAACCCCATGTAATGGTTGCAGAAAACCATACTTGTTATTGATTTT

AAGCAAAGTATCCAT

# PI296341-FR

TTAATCTCTATCCTTTACCAGATTGCCGATCATGTTTACCAAAGATGGAGTTCCCTTTGC

CATGATTTCAAGCCTGGAGGCATTAGATGCGTGAGAGAATAAGGAAAGCCCAAAGAGTAA

CAGCTCAGGAAGGAATAGTCGTGATGACAAGAATCCATGCCAATAACGAGGTTCAAGATC

AAAAAATGCATCAAAAAACCTTCTTGTACCCTTTAGATCCAGCTTCAATAAAATATCCAT

CCCAAAACAGAAAAATTCTCTCTGCCTCCTCCTTTCGATGGGCCATAGATCTTTCCAAAC

TTCAGAGGATATCGCATCACCCCTGAAACGTCCATCTGAACCAAGGCACCGGACTATTGC

ACTAGCAACAATAGGTGCTGCTGCTAGAGTTCTTGCTACCATATATCCAGTTGAAGGGTG

CACCATCCCTGCTGTTCCACCAATTCCAACAACTCTTTGAGGAAGAACTGGCAGCGGTCC

ACCCATTGGAATGACACAATGCTCATCCTCTTCAATGCTCTTCACTTTTATTCCCAAGTG

CTTCAATCTTACCTCCATTCTTTCCTGGATATCGCTCATTTGTAAACCAGGTCGAGCTAC

CAAAGAAGTTTCCTCCAGAAATATCCGATTTGATGAAAAGGGCATTGCATAGAGAAATGT

AGGAATTTTGCTATTTCTCTCCTTCAAAATCATATTGTTATTCAGATGCGAATCTCTCCA

GTCCATAAACACCATCTTGTTAACATCAAATGGATGTTCCTCCACCTCAGCTAAAATCCC

ATAAGCTACCTGGTAGCCTGGATTGTAAGGCTTATCATATTGGACAAGGCATCGAGAGAA

GCCAGTAGCATCAAGAACAATGGCAGCTTGAATGGTCACACCATCATTGCAAATTAACAA

GGATTTGAACTCCTCATGTATAACTTTAATAACTTTAGCTTCATGAAACTTAACACCATT

GGAAATGCATTTCTGCAACATTTTTGACTTGAGTTGCTTTCTATTAACCCTCGCATAAGG

TCGAGCAAGATCTTTTGTTGATTGCTCATTGGTGAACACGACAGCACCAGACCAAGTCGT

GTCGAGACAATCTAGCAAATCCATTGCCTCAAATTCATCCACCCAAACCCCATAATTGTT

GGGCCAAATCAACTTGGGAGATGGGTCAATTGCACAAACTGAAAGCCCTGCCTCTGAAAC

CTGTTGCGCAACAGCAAGCCCTGCTGGGCCGCCTCCCACGACCGCAAGATCGACAACAAG

GCCCTTCGAAGGATCATACATGGGAAGTTCAACCTCAAGATTCTCCTTCTTGGTTCCAGG

--------CCAAAAGAGCACTACTTCTCACATTAAGACAACCCCCTTTCCTCCATTTCAG

ACGACCCTTCCTATGACCAAATCCAAATTCCTGACTCTGAAAC----TACTCCTCACACC

ACTCACTTTTTCCGAAACCCCATGTAATGGTTGCAGAAAACCATACTTGTTATTGATTTT

AAGCAAAGTATCCAT

# RZ-901

TTAATCTCTATCCTTTACCAGATTGCCGATCATGTTTACCAAAGATGGAGTTCCCTTTGC

CATGATTTCAAGCCTGGAGGCATTAGATGCGTGAGAGAATAAGGAAAGCCCAAAGAGTAA

CAGCTCAGGAAGGAATAGTCGTGATGACAAGAATCCATGCCAATAACGAGGTTCAAGATC

AAAAAATGCATCAAAAAACCTTCTTGTACCCTTTAGATCCAGCTTCAATAAAATATCCAT

CCCAAAACAGAAAAATTCTCTCTGCCTCCTCCTTTCGATGGGCCATAGATCTTTCCAAAC

TTCAGAGGATATCGCATCACCCCTGAAACGTCCATCTGAACCAAGGCACCGGACTATTGC

ACTAGCAACAATAGGTGCCGCTGCTAGAGTTCTTGCTACCATATATCCAGTTGAAGGGTG

CACCATCCCTGCTGTTCCACCAATTCCAACAACTCTTTGAGGAAGAACTGGCAGCGGTCC

ACCCATTGGAATGACACAATGCTCATCCTCTTCAATGCTCTTCACTTTTATTCCCAAGTG

CTTCAATCTTACCTCCATTCTTTCCTGGATATCGCTCATTTGTAACCCAGGTCGAGCTAC

CAAAGAAGTTTCCTCCAGAAATATCCGATTTGATGAAAAGGGCATTGCATAGAGAAATGT

AGGAATTTTGCTATTTCTCTCCTTCAAAATCATATTGTTATTCAGATGTGAATCTCTCCA

GTCCATAAACACCATCTTGTTAACATCAAATGGATGTTCCTCCA----------------

-----CTACCTGGTAGCCTGGATTGTAAGGCTTATCATATTGGACAAGGCATCGAGAGAC

GCCAGTGGCATCAAGAACAATGGCAGCTTGAATGGTCACACCATCATTGCAAATTAACAA

GGATTTGAACTCCTCATGTATAACTTTAATAACTTTAGCTTCATGAAACTTAACACCATT

GGAAATGCATTTCTGCAACATTTTTGACTTGAGTTGCTTTCTATTAACCCTCGCATAAGG

TCGAGCAAGATCTTTTGTTGATTGCTCATTGGTGAACACGACAGCACCAGACCAAGTCGT

GTCGAGACAATCTAGCAAATCCATTGCCTCAAATTCATCCACCCAAACCCCATAATTGTT

GGGCCAAATCAACTTGGGAGATGGGTCAATTGCACAAACTGAAAGCCCTGCCTCTGAAAC

CTGTTGCGCAACAGCAA---CTGCTGGGCCGCCTCCCACGACCGCAAGATCGACAACAAG

GCCCTTCGAAGGATCATACATGGGAAGTTCAACCTCAAGATTCTCCTTCTTGGTTTCAGG

AACAAGCTCCAAAAGAGAACTACTTCTCACATTAAGACAACCCCCTTTCCTCCATTTCAG

ACGACCCTTCCTATGACCAAACCCAAATTCCTGACTCTGAAACTTTGTACTCCTCACACC

ACTCACTTTTTCCGAAACCCCATGTAATGGTTGCAGAAAACCATACTTGTTATTGATTTT

AAGTAAAGTATCCAT

# RZ-900

TTAATCTCTATCCTTTACCAGATTGCCGATCATGTTTACCAAAGATGGAGTTCCCTTTGC

CATGATTTCAAGCCTGGAGGCATTAGATGCGTGAGAGAATAAGGAAAGCCCAAAGAGTAA

CAGCTCAGGAAGGAATAGTCGTGATGACAAGAATCCATGCCAATAACGAGGTTCAAGATC

AAAAAATGCATCAAAAAACCTTCTTGTACCCTTTAGATCCAGCTTCAATAAAATATCCAT

CCCAAAACAGAAAAATTCTCTCTGCCTCCTCCTTTCGATGGGCCATAGATCTTTCCAAAC

TTCAGAGGATATCGCATCACCCCTGAAACGTCCATCTGAACCAAGGCACCGGACTATTGC

ACTAGCAACAATAGGTGCCGCTGCTAGAGTTCTTGCTACCATATATCCAGTTGAAGGGTG

CACCATCCCTGCTGTTCCACCAATTCCAACAACTCTTTGAGGAAGAACTGGCAGCGGTCC

ACCCATTGGAATGACACAATGCTCATCCTCTTCAATGCTCTTCACTTTTATTCCCAAGTG

CTTCAATCTTACCTCCATTCTTTCCTGGATATCGCTCATTTGTAACCCAGGTCGAGCTAC

CAAAGAAGTTTCCTCCAGAAATATCCGATTTGATGAAAAGGGCATTGCATAGAGAAATGT

AGGAATTTTGCTATTTCTCTCCTTCAAAATCATATTGTTATTCAGATGTGAATCTCTCCA

GTCCATAAACACCATCTTGTTAACATCAAATGGATGTTCCTCCACCTCAGCTAAAATCCC

ATAAGCTACCTGGTAGCCTGGATTGTAAGGCTTATCATATTGGACAAGGCATCGAGAGAC

GCCAGTGGCATCAAGAACAATGGCAGCTTGAATGGTCACACCATCATTGCAAATTAACAA

GGATTTGAACTCCTCATGTATAACTTTAATAACTTTAGCTTCATGAAACTTAACACCATT

GGAAATGCATTTCTGCAACATTTTTGACTTGAGTTGCTTTCTATTAACCCTCGCATAAGG

TCGAGCAAGATCTTTTGTTGATTGCTCATTGGTGAACACGACAGCACCAGACCAAGTCGT

GTCGAGACAATCTAGCAAATCCATTGCCTCAAATTCATCCACCCAAACCCCATAATTGTT

GGGCCAAATCAACTTGGGAGATGGGTCAATTGCACAAACTGAAAGCCCTGCCTCTGAAAC

CTGTTGCGCAACAGCAAGCCCTGCTGGGCCGCCTCCCACGACCGCAAGATCGACAACAAG

GCCCTTCGAAGGATCATACATGGGAAGTTCAACCTCAAGATTCTCCTTCTTGGTTTCAGG

AACAAGCTCCAAAAGAGAACTACTTCTCACATTAAGACAACCCCCTTTCCTCCATTTCAG

ACGACCCTTCCTATGACCAAACCCAAATTCCTGACTCTGAAACTTTGTACTCCTCACACC

ACTCACTTTTTCCGAAACCCCATGTAATGGTTGCAGAAAACCATACTTGTTATTGATTTT

AAGTAAAGTATCCAT

# Sy-904304

TTAATCTCTATCCTTTACCAGATTGCCGATCATGTTTACCAAAGATGGAGTTCCCTTTGC

CATGATTTCAAGCCTGGAGGCATTAGATGCGTGAGAGAATAAGGAAAGCCCAAAGAGTAA

CAGCTCAGGAAGGAATAGTCGTGATGACAAGAATCCATGCCAATAACGAGGTTCAAGATC

AAAAAATGCATCAAAAAACCTTCTTGTACCCTTTAGATCCAGCTTCAATAAAATATCCAT

CCCAAAACAGAAAAATTCTCTCTGCCTCCTCCTTTCGATGGGCCATAGATCTTTCCAAAC

TTCAGAGGATATCGCATCACCCCTGAAACGTCCATCTGAACCAAGGCACCGGACTATTGC

ACTAGCAACAATAGGTGCCGCTGCTAGAGTTCTTGCTACCATATATCCAGTTGAAGGGTG

CACCATCCCTGCTGTTCCACCAATTCCAACAACTCTTTGAGGAAGAACTGGCAGCGGTCC

ACCCATTGGAATGACACAATGCTCATCCTCTTCAATGCTCTTCACTTTTATTCCCAAGTG

CTTCAATCTTACCTCCATTCTTTCCTGGATATCGCTCATTTGTAACCCAGGTCGAGCTAC

CAAAGAAGTTTCCTCCAGAAATATCCGATTTGATGAAAAGGGCATTGCATAGAGAAATGT

AGGAATTTTGCTATTTCTCTCCTTCAAAATCATATTGTTATTCAGATGTGAATCTCTCCA

GTCCATAAACACCATCTTGTTAACATCAAATGGATGTTCCTCCACCTCAGCTAAAATCCC

ATAAGCTACCTGGTAGCCTGGATTGTAAGGCTTATCATATTGGACAAGGCATCGAGAGAC

GCCAGTGGCATCAAGAACAATGGCAGCTTGAATGGTCACACCATCATTGCAAATTAACAA

GGATTTGAACTCCTCATGTATAACTTTAATAACTTTAGCTTCATGAAACTTAACACCATT

GGAAATGCATTTCTGCAACATTTTTGACTTGAGTTGCTTTCTATTAACCCTCGCATAAGG

TCGAGCAAGATCTTTTGTTGATTGCTCATTGGTGAACACGACAGCACCAGACCAAGTCGT

GTCGAGACAATCTAGCAAATCCATTGCCTCAAATTCATCCACCCAAACCCCATAATTGTT

GGGCCAAATCAACTTGGGAGATGGGTCAATTGCACAAACTGAAAGCCCTGCCTCTGAAAC

CTGTTGCGCAACAGCAAGCCCTGCTGGGCCGCCTCCCACGACCGCAAGATCGACAACAAG

GCCCTTCGAAGGATCATACATGGGAAGTTCAACCTCAAGATTCTCCTTCTTGGTTTCAGG

AACAAGCTCCAAAAGAGAACTACTTCTCACATTAAGACAACCCCCTTTCCTCCATTTCAG

ACGACCCTTCCTATGACCAAACCCAAATTCCTGACTCTGAAACTTTGTACTCCTCACACC

ACTCACTTTTTCCGAAACCCCATGTAATGGTTGCAGAAAACCATACTTGTTATTGATTTT

AAGTAAAGTATCCAT

# Sugarlee

TTAATCTCTATCCTTTACCAGATTGCCGATCATGTTTACCAAAGATGGAGTTCCCTTTGC

CATGATTTCAAGCCTGGAGGCATTAGATGCGTGAGAGAATAAGGAAAGCCCAAAGAGTAA

CAGCTCAGGAAGGAATAGTCGTGATGACAAGAATCCATGCCAATAACGAGGTTCAAGATC

AAAAAATGCATCAAAAAACCTTCTTGTACCCTTTAGATCCAGCTTCAATAAAATATCCAT

CCCAAAACAGAAAAATTCTCTCTGCCTCCTCCTTTCGATGGGCCATAGATCTTTCCAAAC

TTCAGAGGATATCGCATCACCCCTGAAACGTCCATCTGAACCAAGGCACCGGACTATTGC

ACTAGCAACAATAGGTGCCGCTGCTAGAGTTCTTGCTACCATATATCCAGTTGAAGGGTG

CACCATCCCTGCTGTTCCACCAATTCCAACAACTCTTTGAGGAAGAACTGGCAGCGGTCC

ACCCATTGGAATGACACAATGCTCATCCTCTTCAATGCTCTTCACTTTTATTCCCAAGTG

CTTCAATCTTACCTCCATTCTTTCCTGGATATCGCTCATTTGTAACCCAGGTCGAGCTAC

CAAAGAAGTTTCCTCCAGAAATATCCGATTTGATGAAAAGGGCATTGCATAGAGAAATGT

AGGAATTTTGCTATTTCTCTCCTTCAAAATCATATTGTTATTCAGATGTGAATCTCTCCA

GTCCATAAACACCATCTTGTTAACATCAAATGGATGTTCCTCCACCTCAGCTAAAATCCC

ATAAGCTACCTGGTAGCCTGGATTGTAAGGCTTATCATATTGGACAAGGCATCGAGAGAC

GCCAGTGGCATCAAGAACAATGGCAGCTTGAATGGTCACACCATCATTGCAAATTAACAA

GGATTTGAACTCCTCATGTATAACTTTAATAACTTTAGCTTCATGAAACTTAACACCATT

GGAAATGCATTTCTGCAACATTTTTGACTTGAGTTGCTTTCTATTAACCCTCGCATAAGG

TCGAGCAAGATCTTTTGTTGATTGCTCATTGGTGAACACGACAGCACCAGACCAAGTCGT

GTCGAGACAATCTAGCAAATCCATTGCCTCAAATTCATCCACCCAAACCCCATAATTGTT

GGGCCAAATCAACTTGGGAGATGGGTCAATTGCACAAACTGAAAGCCCTGCCTCTGAAAC

CTGTTGCGCAACAGCAAGCCCTGCTGGGCCGCCTCCCACGACCGCAAGATCGACAACAAG

GCCCTTCGAAGGATCATACATGGGAAGTTCAACCTCAAGATTCTCCTTCTTGGTTTCAGG

AACAAGCTCCAAAAGAGAACTACTTCTCACATTAAGACAACCCCCTTTCCTCCATTTCAG

ACGACCCTTCCTATGACCAAACCCAAATTCCTGACTCTGAAACTTTGTACTCCTCACACC

ACTCACTTTTTCCGAAACCCCATGTAATGGTTGCAGAAAACCATACTTGTTATTGATTTT

AAGTAAAGTATCCAT
